# Gain-of-function mutant of movement protein allows systemic transport of a defective tobacco mosaic virus

**DOI:** 10.1016/j.isci.2022.105486

**Published:** 2022-11-03

**Authors:** Phu-Tri Tran, Mi-Sa Vo Phan, Vitaly Citovsky

**Affiliations:** 1 Department of Biochemistry and Cell Biology, State University of New York, Stony Brook, NY 11794-5215, USA

**Keywords:** Virology, Cell biology, Molecular plant pathology

## Abstract

Functional compensation in response to gene dysfunction is a fascinating phenomenon that allows mutated viruses to regain the capabilities of their wild-type parental strains. In this study, we isolated mutants of tobacco mosaic virus capable of CP-independent systemic movement. These gain-of-function mutants lacked the 16 C-terminal amino acids of the movement protein (MP). Whereas this deletion did not affect the cell-to-cell movement of MP, it dramatically enhanced the viral genomic RNA levels and MP accumulation within the infected cells and altered the subcellular localization of MP from exclusively plasmodesmata (PD) to both PD and plasma membrane. The adapted defective virus suppressed the expression of the ethylene pathway and phloem-associated resistance factors in the inoculated leaves. These findings demonstrate the potential for plant viral MPs to gain a new function that allows viral genomes to move systemically in the absence of the natural viral factor that mediates this spread.

## Highlights

• TMVΔCPmutMP lacking 16 C-terminal residues of MP systemically moves in *N*. *benthamiana*
• The mutation of MP did not change its cell-to-cell moment but subcellular localization
• The mutation of MP enhanced the viral genomic RNA levels and accumulation of MP
• TMVΔCPmutMP locally suppressed expression of ET pathway and phloem-associated resistance

## Graphical abstract

**Figure:**
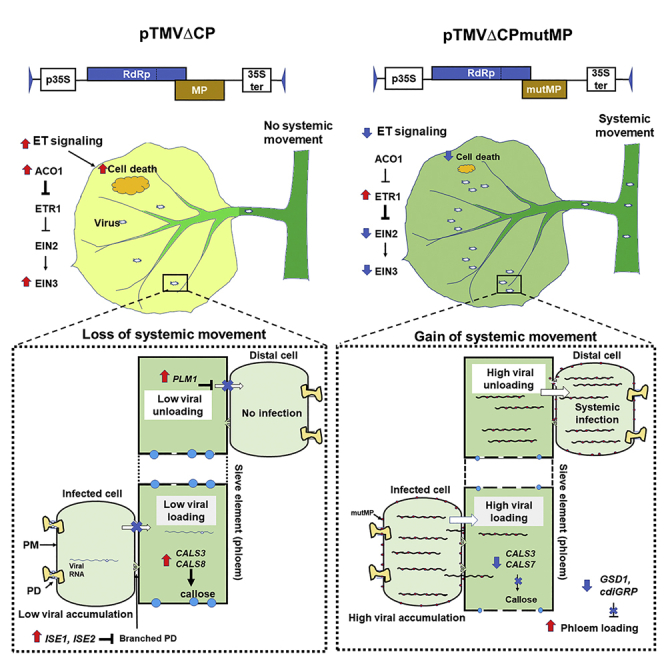

## Introduction

Functional compensation in response to gene dysfunction is a common phenomenon in many organisms. The fitness losses caused by gene mutations can be buffered or compensated by genetic redundancy in which no or little effect of the mutations is evident due to the same or similar function of one or several other genes (Rutter et al., 2017). This mechanism is rare in RNA viruses, because of their need for compression of genome size leading to virtually no duplicated sequences, fewer control elements, and overlapping reading frames (Krakauer, 2000; Simon-Loriere and Holmes, 2013). Instead, many viruses have evolved the capacity for high mutation rates, leading to numerous variant genomes (Elena et al., 2006); some of these mutations are compensatory, and they often result in the recovery of the wild-type-like phenotypes (Rokyta et al., 2002; Seki and Matano, 2012).

Most plant viruses spread within their hosts by a bimodal process, i.e., by local and systemic movement. Local infection is mediated by cell-to-cell movement, in which the virus moves from the infected to healthy cells through plasmodesmata (PD), the plant intercellular connections (Tabassum and Blilou, 2022). Once the local infection reaches the plant vascular system, systemic movement ensues to spread the viral infection to distant plant tissues (Kappagantu et al., 2020). Tobacco mosaic virus (TMV)—the first virus discovered and, since then, one of the paradigms for plant viruses—has a small, 6.4 kb, positive-sense RNA genome encoding two overlapping replicases, a cell-to-cell movement protein (MP), and a coat protein (CP) (Scholthof et al., 1999). MP is thought to mediate the local spread of the virus whereas CP is absolutely required for the systemic movement (Hipper et al., 2013). Indeed, in absence of CP, the virus can only replicate and spread locally by the cell-to-cell movement mechanism (Hilf and Dawson, 1993; Ryabov et al., 1999; Venturuzzi et al., 2021).

But does MP have a latent capacity also to facilitate the systemic movement? Here, we identified two spontaneous TMV mutants capable of systemic movement in absence of CP. These gain-of-function mutations were localized to the MP sequence and represented a deletion of 16 C-terminal amino acids of the protein. Whereas this gain-of-function mutation (mutMP) did not significantly affect the cell-to-cell movement of MP, it dramatically enhanced the viral genomic RNA levels, MP accumulation within the infected cells, and altered the subcellular localization of MP from exclusively PD to both PD and plasma membrane. The virus coding for mutMP suppressed the expression of the ethylene pathway and phloem-associated resistance factors in inoculated leaves. Thus, our observations uncover the potential capacity of MP, which normally effects local viral movement, to compensate for the loss of CP and mediate systemic movement of the viral genomes.

## Results

### Identification of the gain-of-function viral mutants with restored systemic movement

**Figure 1:**
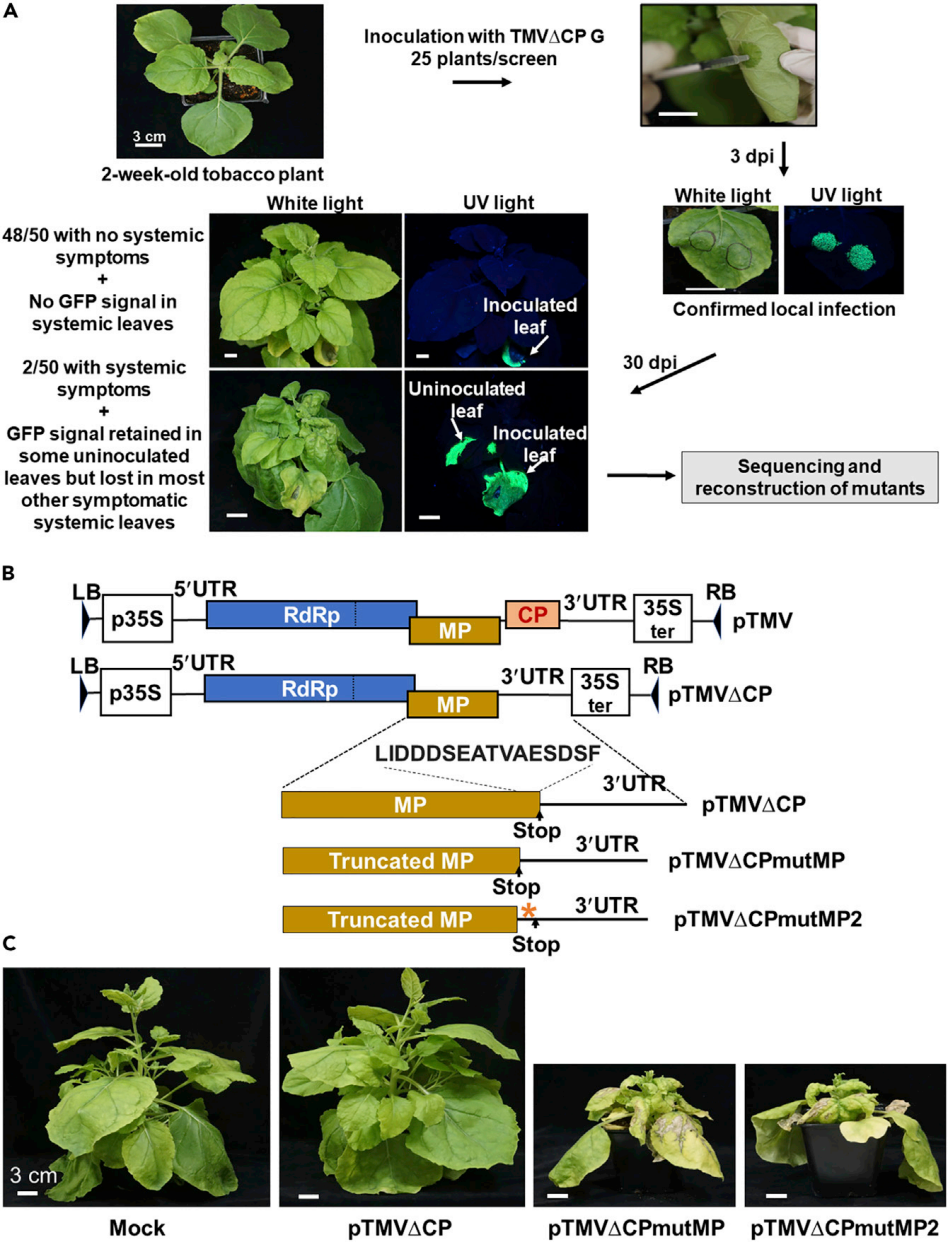
Identification and reconstruction of the TMVΔCPmutMP mutants (A) Experimental screening system to identify TMVΔCP mutants capable of systemic movement. Two-week-old *N*. *benthamiana* plants were agroinfiltrated with pTMVΔCP G which expresses GFP, a viral movement marker. Local infection of the viral vector was confirmed at 3 dpi by expression of GFP as detected under UV light. The inoculated plants were monitored until 30 dpi to identify plants that developed systemic viral disease symptoms. RNA was extracted from the tissues from the plant with the most severe symptoms, followed by cDNA synthesis, sequencing, and re-construction of the recovery mutant. (B) Schematic diagrams of the binary vectors pTMV, pTMVΔCP, pTMVΔCPmutMP, and pTMVΔCPmutMP2 with the indicated locations of the Agrobacterium T-DNA left border (LB) and right border (RB) sequences, and the CaMV 35S promoter (p35S) and terminator (35S ter). The genome of TMV contains the indicated untranslated regions (UTRs) and open reading frames of the RNA-dependent RNA polymerase (RdRp), movement protein (MP), and coat protein (CP). The coding sequence for CP is absent from the genome of pTMVΔCP. Stop codons that terminate translation of MPs in the three viral mutants, pTMVΔCP, pTMVΔCPmutMP, and pTMVΔCPmutMP2, are indicated. The location and sequence of the 16-aa C-terminal region of MP which is present in pTMVΔCP but absent in pTMVΔCPmutMP and pTMVΔCPmutMP2 is indicated. Asterisk indicates the location of the new 14-aa C-terminal sequence gained by pTMVΔCPmutMP2 from the native 3′UTR as a result of the corresponding relocation of the stop codon due to the mutMP2 mutation. (C) *N*. *benthamiana* plants two weeks after inoculation with the MMA buffer (Mock) or with the reconstructed infectious clones pTMVΔCP, pTMVΔCPmutMP, and pTMVΔCPmutMP2. Only plants inoculated with pTMVΔCPmutMP and pTMVΔCPmutMP2 developed the systemic viral disease symptoms.

To identify an adaptive TMV mutant with capability for systemic movement, we inoculated by agroinfiltration in *Nicotiana benthamiana* plants with an infectious TMV clone, pTMVΔCP G, that lacks CP—and thus is unable to spread systemically—and expresses free GFP to facilitate detection of viral spread. Confirming the inability of TMVΔCP G to move systemically, in most of these inoculations (96%), the virus did not spread beyond the inoculated leaves. However, we identified two independently inoculated plants that developed relatively severe systemic symptoms of the viral disease, e.g., leaf curling, shoot stunting, and leaf distortion, in their uninoculated, apical leaves (Figure 1A). Interestingly, the occurrence of the disease symptoms in the systemic leaves was much more prevalent than the detectible accumulation of the GFP signal in the same leaves (Figure 1A), suggesting the loss of GFP expression during adaptation. Sequence analyses of the viral genomic region that includes the GFP expression cassette from both systemically moving isolates revealed the complete loss of the GFP coding sequence and of the sequence LIDDDSEATVAESDSF—the 16 C-terminal amino acid residues of MP (16-aa C-terminus) (Figures 1B and S1A). These spontaneous mutants were designated TMVΔCPmutMP and TMVΔCPmutMP2, both of which lost the 16-aa C-terminus but the TMVΔCPmutMP2 also gained 14 new residues from the native 3′-untranslated region of the TMV genome (Figure 1B, asterisk). On the genomic RNA level, the mutMP mutation in TMVΔCPmutMP did not interfere with most of the *cis*-acting elements that remained in the parental strain TMVΔCP, i.e., the MP subgenomic promoter, the 3′UTR upstream pseudoknot domain, and the 3′UTR tRNA-like structure (Figure S1B) (Grdzelishvili et al., 2000; van Belkum et al., 1985; Zeenko et al., 2002). The only *cis*-acting element affected by mutMP was the CP subgenomic promoter (Grdzelishvili et al., 2000), which most likely is not biologically relevant for TMVΔCP or TMVΔCPmutMP because these virus variants have no coding sequences to be transcribed from this promoter (Figure S1B).

To examine whether these MP mutants indeed represent the causative agents of the systemic symptoms, we reconstructed each of them in a binary vector and evaluated the infectivity of the resulting clones, designated pTMVΔCPmutMP and pTMVΔCPmutMP2, in *N*. *benthamiana*. One week after inoculation, both mutants consistently developed severe systemic symptoms (Figure 1C). These observations indicate that deletion of the 16-aa C-terminus of MP is sufficient to confer systemic movement ability on the CP-defective TMV, suggesting that the 16-aa C-terminus deletion, termed mutMP, represents a gain of function mutation. Thus, we used the pTMVΔCPmutMP mutant for further characterization.

### Effects of the mutMP mutation on the cell-to-cell movement and subcellular localization of MP

**Figure 2:**
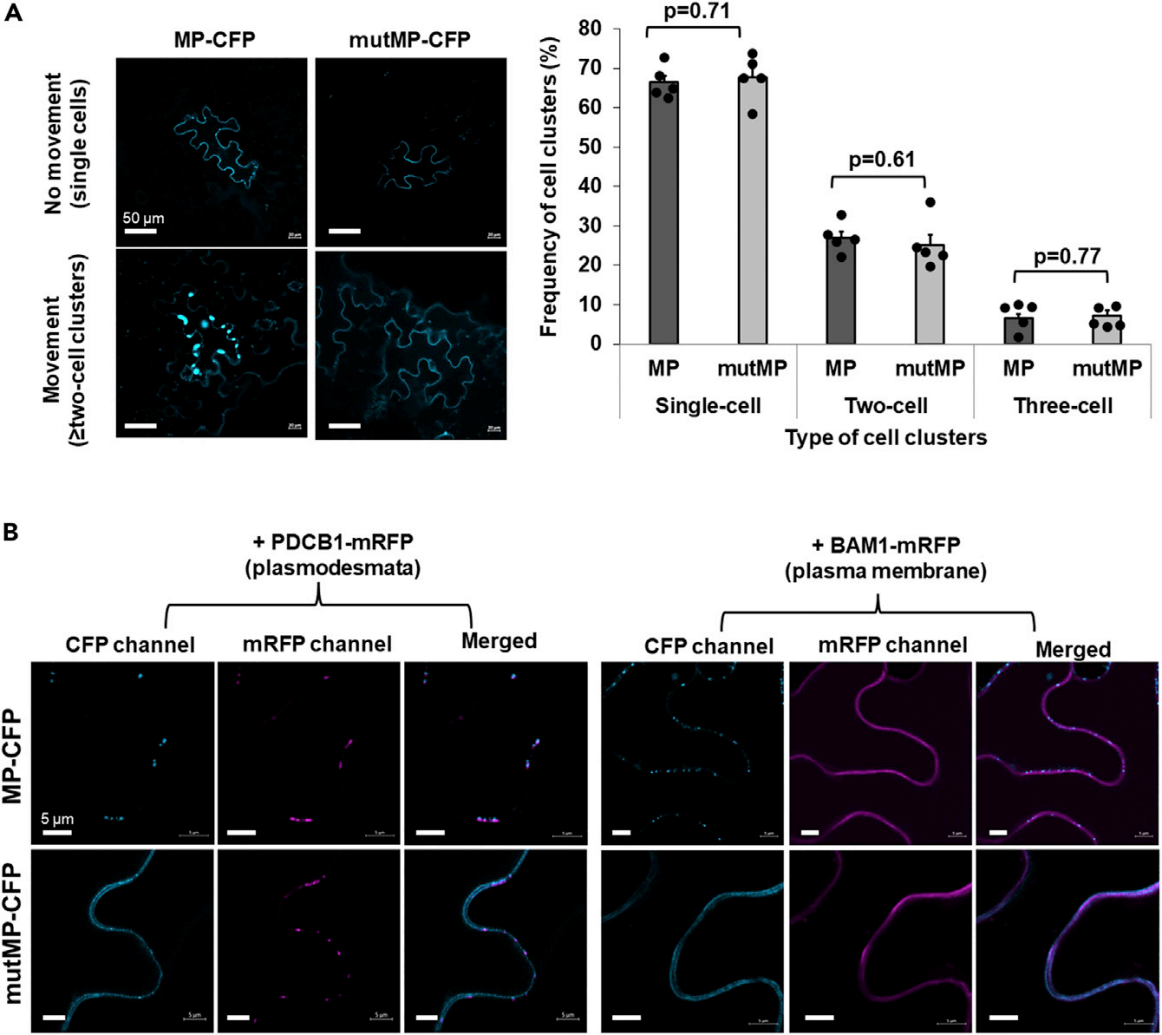
mutMP cell-to-cell movement and subcellular localization in *N*. *benthamiana* (A) Cell-to-cell movement of mutMP. Left panel. Representative images of the cell-to-cell movement of MP-CFP or mutMP-CFP visualized under a confocal microscope. Single-cell or multi-cell clusters represent the absence or presence of cell-to-cell movement, respectively. Right panel. Frequency of cell clusters at 2 dpi with constructs expressing MP-CFP (MP) or mutMP-CFP (mutMP). More than 40 independent cell clusters containing the CFP signal from each of 5 different plants inoculated with the tested constructs were counted for each experimental condition. The extent of cell-to-cell movement was scored as the frequency of the cell clusters composed of ≥2 cells/cluster. The resulting values were expressed as mean ± SE (n = 5). Individual data points are represented by black dots and their numerical values are listed in Table S2. Differences between mean values assessed by the two-tailed t-test are statistically significant if the p value is < 0.05. (B) Subcellular localization of mutMP. Left panel. Co-expression of MP-CFP or mutMP-CFP with the plasmodesmata marker PDCB1-mRFP. Right panel. Co-expression of MP-CFP or mutMP-CFP with the plasma membrane maker BAM1-mRFP. Subcellular localization of the co-expressed proteins was analyzed by confocal microscopy. All images are single confocal sections.

TMV MP itself can move between plant cells without the presence of the viral RNA (Crawford and Zambryski, 2000). To assess whether the mutMP mutation alters this function, the wild-type MP and mutMP were tagged with CFP and transiently expressed in *N*. *benthamiana* leaf epidermis following agroinfiltration. Expression of both MP variants produced a CFP signal in single-cell clusters at 36 h after transfection. Two days after transfection, the cell-to-cell movement of MP was observed as the appearance of ≥2 cell clusters that accumulated CFP (Figure 2A, left panel). Quantification of the numbers of such clusters did not detect statistically significant differences between the wild-type MP and mutMP in the cell-to-cell movement frequency (Figure 2A, right panel). Thus, mutMP produced no detectable effects on the cell-to-cell movement of the MP protein.

We then examined whether mutMP affects the subcellular localization of MP. To this end, we transiently co-expressed CFP-tagged MP and mutMP in *N*. *benthamiana* leaf epidermal cells with different fluorescently tagged subcellular localization markers, e.g., PDCB1-mRFP which represents a PD marker (Figures 2B and S2A) or BAM1-mRFP which represents a plasma membrane marker (Figures 2B and S2B). As expected, MP-CFP exhibited a predominantly punctate appearance diagnostic of PD (Yuan et al., 2018) and colocalized with PDCB1-mRFP but not with BAM1-mRFP (Figures 2B and S2A, upper rows). In contrast, mutMP-CFP was located at both PD and the plasma membrane, colocalizing with their respective marker proteins (Figures 2B, S2A, and S2B, lower rows). We did not observe colocalization of MP and mutMP with the ER (Figure S2C), cell wall (Figure S2D), or nucleocytoplasmic markers (Figure S2). These observations suggest that the mutMP mutation broadened the MP subcellular localization, extending it from solely PD to both PD and the plasma membrane.

### Effects of the mutMP mutation on the accumulation of the total viral RNA and MP in the inoculated and systemic leaves

**Figure 3:**
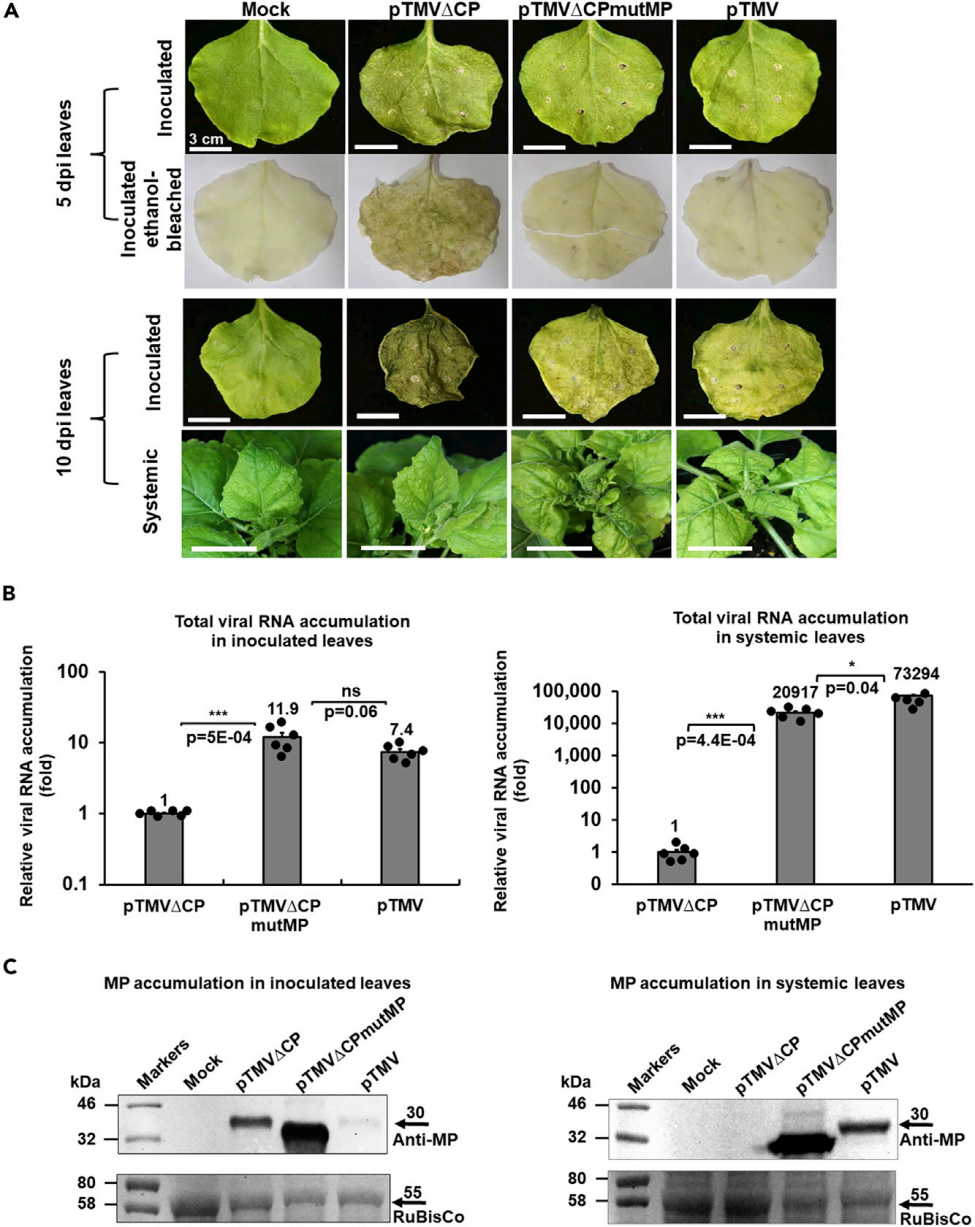
Viral disease symptoms and relative accumulation of the total viral RNA and the MP protein in *N*. *benthamiana* plants inoculated with the wild-type TMV and TMVΔCP and TMVΔCPmutMP mutants (A) Symptom development in the pTMVΔCP, pTMVΔCPmutMP, and pTMV-inoculated plants. Plants inoculated with the MMA buffer (Mock) were used as a negative control. Top panel. Inoculated leaves at 5 dpi; tissue necrosis was visualized by ethanol-bleaching. Bottom panel. Systemic and inoculated leaves at 10 dpi. (B) Relative accumulation of the total viral RNA in the plants inoculated with pTMVΔCP, pTMVΔCPmutMP, or pTMV. The qRT-PCR analysis was performed using the MP-specific primers (see Table S1). Left panel. Inoculated leaves at 4 dpi. Right panel. Systemic leaves at 14 dpi. Viral RNA accumulation in leaves inoculated with pTMVΔCP was set as 1.0. The resulting values were expressed as mean ± SE (n = 6). Individual data points are represented by black dots and their numerical values are listed in Table S3. Differences between mean values assessed by the two-tailed t-test are statistically significant for the p-values ∗p < 0.05, ∗∗p < 0.01, ∗∗∗p < 0.001, and ∗∗∗∗p < 0.0001; p ≥ 0.05 are not statistically significant (ns). (C) Western blot analysis of the accumulation of MP produced by pTMVΔCP, pTMVΔCPmutMP, and pTMV. Left panel. Inoculated leaves at 4 dpi. Right panel. Systemic leaves at 14 dpi. Mock, plants inoculated with the MMA buffer. Molecular markers are indicated in thousands of daltons on the left. Top panels, western blots using anti-MP antibodies. The expected TMV MP (GenBank accession number P03583) electrophoretic mobility of 30 kDa is indicated by an arrow on the right. Lower panels, Ponceau S staining of RuBisCo to assess relative loading of samples. The expected RuBisCo (ribulose-1,5-bisphosphate carboxylase/oxygenase) large subunit electrophoretic mobility of 55 kDa is indicated by an arrow on the right. The original western blots are shown in Figure S7.

Does mutMP affect the accumulation of viral RNA and MP in plant tissues? We investigated this question by quantifying the accumulation of the total viral RNA in the leaves inoculated with pTMVΔCPmutMP, its parental strain pTMVΔCP, and the wild-type virus as a positive control. The total viral RNA comprises the full-length, genomic RNA of the virus as well as its subgenomic RNA transcribed from the subgenomic promoters (Figure S1A); thus, we performed the qRT-PCR analysis using the primers specific for the *MP* sequence (Table S1) which detect both the genomic viral RNA and the MP subgenomic RNA species (Figure S1B). To avoid the interference of local response at ≥5 days post-inoculation (dpi) with pTMVΔCP (Figure 3A), these experiments were performed at 4 dpi. Figure 3B (left panel) shows that, relative to pTMVΔCP, inoculation with pTMVΔCPmutMP resulted in ca. 12-fold higher levels of the total viral RNA, comparable to the amounts of the total viral RNA accumulated in the pTMV-inoculated leaves. At the same infection time point in the same leaves, our Western blot analysis revealed substantially higher levels of MP accumulation in the pTMVΔCPmutMP-inoculated leaves as compared both to the pTMVΔCP and pTMV inoculations (Figure 3C, left panel), suggesting the mutMP mutation may increase the stability of MP, known to undergo rapid turnover in the infected cells (Szécsi et al., 1999).

We then considered the effect of the mutMP mutation on the process of systemic infection by analyzing the levels of the total viral RNA and MP levels in the systemic leaves at 14 dpi (Figures S3). Figure 3B (right panel) shows that, as expected, the total viral RNA of pTMVΔCP, which does not move systemically, was detected in the systemic leaves only at the background level whereas the RNA of pTMV, also as expected, accumulated to very high levels. The total viral RNA of pTMVΔCPmutMP accumulated in the systemic leaves but at levels 3–4 times lower than those of pTMV (Figure 3B, right panel). This difference may be due to the presence of CP in systemic leaves infected by the wild-type virus which may associate with and protect the viral RNA molecules (Ivanov and Mäkinen, 2012) whereas TMVΔCPmutMP does not encode CP. The Western blot analysis of the same leaves showed that infection with pTMVΔCPmutMP resulted in a very substantial accumulation of MP which even exceeds that produced by the systemic pTMV infection. Obviously, no MP was observed in the systemic leaves of the plants inoculated with pTMVΔCP that does not spread systemically (Figure 3C, right panel).

Next, we focused our analysis on the genomic viral RNA, without the subgenomic species. To this end, we utilized the primers specific for *RdRp* (RNA-directed RNA polymerase) (Table S1) which detect only the genomic viral RNA species (Figure S1B). Similarly, to the accumulation of the total viral RNA (see Figure 3B), the accumulation of the pTMVΔCPmutMP genomic RNA in the inoculated leaves was 10-fold higher than that of pTMVΔCP and comparable to that of pTMV (Figure S1C, left panel). Also, in the systemic leaves, the accumulation pattern of the genomic viral RNA mirrored the accumulation of the total viral RNA (compare right panels in Figures S1C and 3B) although we did not observe statistically significant differences between pTMVΔCPmutMP and pTMV. Thus, the increase in viral RNA accumulation in the pTMVΔCPmutMP-infected local and systemic tissues is general and does not reflect a possible specific increase in the accumulation of the subgenomic MP RNA.

Besides the MP subgenomic promoter, the TMV genome contains the CP subgenomic promoter, although the CP gene itself is absent in pTMVΔCPmutMP and in its parental pTMVΔCP strain. (Figure S1B) Thus, to assess the possible effect of the mutMP mutation on the accumulation of transcripts produced from the CP subgenomic promoter, we analyzed the amounts of 3′UTR RNA which is located downstream from the CP gene (Figure S1B) and derives largely from the activity of the CP subgenomic promoter (Grdzelishvili et al., 2000). Figure S1D shows that the inoculated leaves infected with pTMVΔCPmutMP accumulated lower amounts of the 3′UTR-specific viral RNA than the leaves infected with pTMV or pTMVΔCP. The most striking difference was observed in the systemic leaves where the infection with pTMV produced ca. 30-fold more 3′UTR-specific viral RNA than the infection with pTMVΔCPmutMP (Figure S1D, right panel). These observations indicate that the mutMP mutation had indeed compromised the activity of the CP subgenomic promoter, with the residual 3′UTR-specific transcript most likely generated from the genomic and MP subgenomic promoters.

Finally, our analyses of the viral RNA and the MP protein accumulation in the systemic leaves were confirmed and extended by analyzing the content of the viral RNA-MP complexes. TMV MP is well known to associate with the single-stranded nucleic acids (Brill et al., 2000; Citovsky et al., 1990, 1992). Thus, we immunopurified MP from the systemic leaves and analyzed it for the presence of the MP-associated viral RNA relative to the total MP accumulated in the infected cells (Figure S4A). Figure S4B shows that the pTMVΔCPmutMP-infected systemic leaves accumulated ca. 40-fold higher amounts of the viral RNA than the leaves infected by pTMV, consistent with much higher amounts of MP found in these leaves (see Figure 3C, right panel). Potentially, CP of pTMV can encapsidate the viral RNA, thereby reducing its association with MP, whereas this RNA sequestration does not occur with the pTMVΔCPmutMP infection where CP does not exist.

### Suppression of the ethylene signaling factors by TMVΔCPmutMP

Incompatible interactions between viruses and plants often culminate with a hypersensitive reaction or cell death-like response at the infection loci (Garcia-Ruiz, 2019). Indeed, the local infection of pTMVΔCP, but not by pTMVΔCPmutMP or pTMV, in *N*. *benthamiana* resulted in tissue necrosis that led to partial or complete necrosis and shedding of the inoculated leaves (Figure 3A) but not of the systemic leaves (Figures 3A and S3). This reaction most likely represents the antiviral response of the plant, and this response was less efficient against pTMVΔCPmutMP and pTMV than against pTMVΔCP. That the main functional difference between these viral strains is their capacity or the lack thereof to move systemically suggests that it is the viral factor that allows the systemic movement, i.e., mutMP or CP, that counteracts the resistance. Thus, we examined whether mutMP—which suppressed the necrosis response and helped pTMVΔCPmutMP escape the inoculated leaves—can suppress the antiviral signaling of the host. The results of these experiments with pTMVΔCPmutMP and pTMV were compared to pTMVΔCP, the parental strain of pTMVΔCPmutMP, and, therefore, the point of reference for the effects of the mutMP mutation.

**Figure 4:**
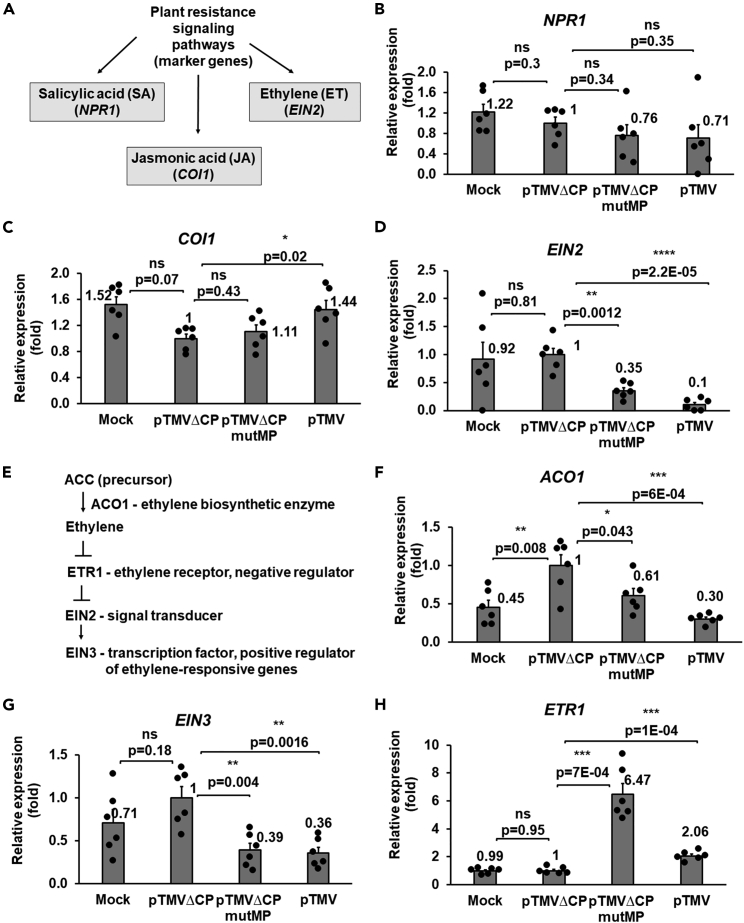
Effects of TMVΔCPmutMP on the expression of resistance signaling genes in *N*. *benthamiana* (A) A schematic diagram depicts marker genes for the salicylic acid (SA), jasmonic acid (JA), and ethylene (ET) signaling pathways involved in plant resistance against pathogens. (B–D) Expression of *NPR1*, *COI1*, and *EIN2* in the plants inoculated with the MMA buffer (Mock), pTMVΔCP, pTMVΔCPmutMP, or pTMV at 4 dpi in the inoculated leaves. (E) A schematic diagram depicts several key regulators of the ET signaling pathway. Synthesis of ethylene from its precursor ACC is catalyzed by ACO1; ethylene inhibits the activity of its receptor ETR1 which is a negative regulator of EIN2, an ethylene signal transducer; EIN2 activates the transcription factor EIN3 which is a positive regulator of ethylene-responsive genes. (F–H) Expression of *ACO1*, *EIN3*, and *ETR1* in the plants inoculated with the MMA buffer (Mock), pTMVΔCP, pTMVΔCPmutMP, or pTMV at 4 dpi in the inoculated leaves. The expression level in leaves inoculated with pTMVΔCP was set as 1.0. The resulting values were expressed as mean ± SE (n = 6). Individual data points are represented by black dots and their numerical values are listed in Tables S4 and S5. Differences between mean values assessed by the two-tailed t-test are statistically significant for the p-values ∗p < 0.05, ∗∗p < 0.01, ∗∗∗p < 0.001, and ∗∗∗∗p < 0.0001; p ≥ 0.05 are not statistically significant (ns).

For TMV, signaling pathways mediated by salicylic acid (SA), jasmonic acid (JA), and ethylene (ET) are known to regulate the resistance of *N*. *benthamiana* to the virus (Zhu et al., 2014, 2022) (Figure 4A). Figures 4B and 4C show that the expression levels of *NPR1* and *COI1*—key genes of the SA and JA pathways, respectively—were not significantly altered in tissues infected by pTMVΔCPmutMP relative to pTMVΔCP at 4 dpi, i.e., before the onset of necrosis. However, the expression of *EIN2*, one of the key genes of the ET signaling pathway, was strongly and in a statistically significant fashion suppressed by both pTMVΔCPmutMP and pTMV in comparison to pTMVΔCP (Figure 4D).

These effects also were observed for several other ET signaling pathway-related genes (Figure 4E), i.e., *ACO1* and *EIN3*. Specifically, pTMVΔCPmutMP and pTMV exhibited various and statistically significant degrees of suppression of the *ACO1* and *EIN3* genes relative to pTMVΔCP (Figures 4F and 4G). Consistent with their suppressive effects on positive regulators/components of the ET signaling pathway, both pTMVΔCPmutMP and pTMV induced the expression of *ETR1*, a negative regulator of the ET signaling, as compared to pTMVΔCP (Figure 4H). Figure 4 also shows that all tested genes were expressed, to varying degrees, in tissues that were “mock”-inoculated with the buffer and have not undergone agroinfiltration and have not encountered the virus. Together, these data indicate that pTMVΔCPmutMP suppressed several key factors of the ET pathway, but not of the SA or JA pathways, within the inoculated leaves.

### Suppression of the phloem loading/unloading factors by TMVΔCPmutMP

**Figure 5:**
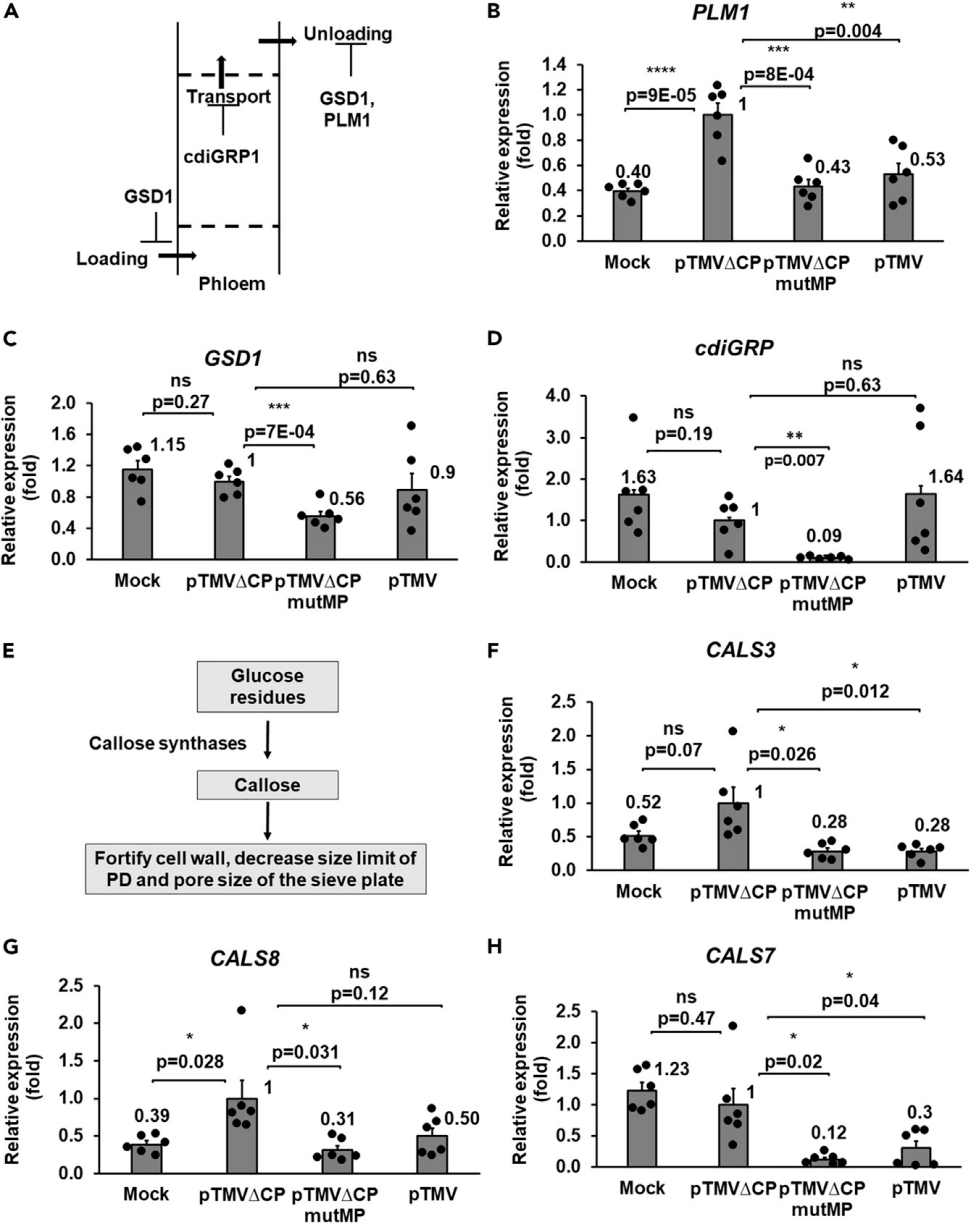
TMVΔCPmutMP suppresses host factors involved in phloem loading/unloading in *N*. *benthamiana* (A) A schematic diagram of phloem loading and unloading controlled by negative regulators GSD1, cdiGRP, and PLM1. (B–D) Expression of phloem-specific *PLM1*, *GSD1*, and *cdiGRP* in the plants inoculated with the MMA buffer (Mock), pTMVΔCP, pTMVΔCPmutMP, or pTMV at 4 dpi in the inoculated leaves. (E) A schematic diagram of a simplified biochemical pathway of callose deposition mediated by callose synthases. (F–H) Expression of phloem-specific *CAL3*, *CAL7*, *and CALS8* in the plants inoculated with the MMA buffer (Mock), pTMVΔCP, pTMVΔCPmutMP, or pTMV at 4 dpi in the inoculated leaves. The expression level in leaves inoculated with pTMVΔCP was set as 1.0. The resulting values were expressed as mean ± SE (n = 6). Individual data points are represented by black dots and their numerical values are listed in Tables S5–S7. Differences between mean values assessed by the two-tailed t-test are statistically significant for the p values ∗p < 0.05, ∗∗p < 0.01, ∗∗∗p < 0.001, and ∗∗∗∗p < 0.0001; p ≥ 0.05 are not statistically significant (ns).

Loading into the phloem and unloading into systemic tissues are the key steps of the systemic spread of the virus after it reaches the vascular system of the inoculated organ. The processes of entry into and egress from the phloem involve several host genes, such as *PLM1*, *GSD1*, and *cdiGRP* (Figure 5A) (Gui et al., 2014; Ueki and Citovsky, 2002; Yan et al., 2019). We compared the effects of the systemic movement-capable pTMVΔCPmutMP and pTMV viruses on the expression of these genes relative to the systemic movement-incapable pTMVΔCP virus. *PLM1* encodes a sphingolipid biosynthetic enzyme, the absence of which increases the phloem unloading (Yan et al., 2019). Figure 5B shows that pTMVΔCPmutMP and pTMV did not significantly alter the expression of *PLM1* observed in the absence of the virus, but pTMVΔCP strongly activated it. *GSD1* encodes a remorin-like protein, the enhanced expression of which impairs transport into the phloem (Gui et al., 2014). Figure 5C shows that pTMVΔCPmutMP suppressed the *GSD1* expression relative to pTMVΔCP whereas pTMV had no statistically significant effect. Finally, *cdiGRP* codes for a glycine-rich protein that inhibits systemic movement of tobamoviruses (Ueki and Citovsky, 2002), and, again, only pTMVΔCPmutMP, but not pTMV, suppressed the expression of *cdiGRP* with statistical significance relative to pTMVΔCP (Figure 5D).

Callose production by callose synthases and its deposition in the sieve plate is another factor that can interfere with the systemic movement of plant viruses (Wang et al., 2021) (Figure 5E). Thus, we determined the expression levels of three callose synthase genes, *CALS3*, *CALS7*, and *CALS8*, at 4 dpi in the leaves inoculated with pTMVΔCPmutMP, pTMVΔCP, or pTMV. These experiments showed that pTMVΔCP induced the expression of *CALS3* (Figure 5F) and *CALS8* (Figure 5G) and did not significantly affect the expression of *CALS7* (Figure 5H). Conversely, pTMVΔCPmutMP suppressed the expression of *CALS3*, *CALS8*, and *CALS7* by 3.5-fold, 3.2-fold, and 8.3-fold relative to pTMVΔCP, respectively, in a statistically significant fashion (Figures 5F–5H). pTMV also inhibited the expression of *CALS3* and *CALS7* (Figures 5F and 5H) but did not affect the expression of *CALS8* with statistical significance relative to pTMVΔCP (Figure 5G). Taken together, these results suggest that mutMP may act to suppress numerous and diverse host factors that negatively affect the phloem loading/unloading.

Plant virus infection considerably affects the structure and morphology of many cellular organelles (Laliberté and Sanfaçon, 2010) and alters the structure of PD and thus the intercellular traffic (Azim and Burch-Smith, 2020; Brunkard et al., 2013). The native structure and permeability of PD, in turn, depend on several endogenous plant factors, including the mitochondrial *ISE1* and chloroplastic *ISE2* genes, the downregulation of which increases intercellular transport and promotes the formation of branched PD (Burch-Smith and Zambryski, 2010). Because this type of PD is enriched at the companion cell/phloem sieve element boundary (Miras et al., 2022) which the systemically moving TMV must cross, we analyzed the potential effects of pTMVΔCPmutMP, pTMVΔCP, or pTMV inoculation on the expression of *ISE1* and *ISE2* in the inoculated leaves. Figure S5 shows that both genes were expressed at significantly higher levels in pTMVΔCP-inoculated leaves whereas inoculation with pTMVΔCPmutMP or pTMV had no statistically significant effects on the expression of *ISE1* (Figure S5A) and *ISE2* (Figure S5B). These results suggest different modes of regulation of the *ISE1* and *ISE2* expression upon incompatible (pTMVΔCP) or compatible (pTMVΔCPmutMP and pTMV) interactions between the plant host and the invading viral pathogen. Similarly, Figures 4 and 5 show the basal expression levels for all tested genes in “mock” inoculated tissues.

## Discussion

### Lack of CP drives the evolution of the TMV genome and reveals the potential new function of MP

The members of the genus *Tobamovirus* known today have co-evolved with their hosts, and, through natural selective pressure, the viral proteins have gained and perfected the specific biological functions essential for their fitness in specific hosts (Gibbs, 1999). On the other hand, like most RNA viruses, the TMV replication is error prone which can lead to diverse variations in protein functions (Elena et al., 2006). Deletion of CP from the viral genome creates a novel and powerful selective pressure for the evolution of the defective TMV genome which has lost the capacity for systemic movement but remained able to replicate and accumulate potential gain-of-function mutations. Taking advantage of this unique experimental system, we demonstrated that the CP-deficient virus can evolve and recover the ability for systemic infection by a gain-of-function modification of its MP, mutMP, which was achieved through the loss of its 16 C-terminal amino acids. Normally, MP is not a direct participant in the systemic movement of the virus per se (Hilf and Dawson, 1993). What is the molecular and functional basis for this newly acquired systemic movement capacity of this gain-of-function MP mutant?

The 16-aa C-terminal domain of MP may serve as a negative regulator for the constitutive presence of MP in the host cells. Indeed, while the TMV RNA-dependent RNA polymerase (RdRp) and CP proteins are expressed throughout the course of infection, the presence of MP is only transient (Szécsi et al., 1999; Watanabe et al., 1984) most likely due to its destruction by the 26S proteasome (Reichel and Beachy, 2000). Removal of this C-terminal domain substantially increases the accumulation of the resulting mutMP protein in both inoculated and systemic leaves, suggesting that mutMP may, at least in part, escape proteasomal degradation and that the deleted MP domain may contain post-translational modifications that signal degradation. Indeed, a deep-learning-based motif prediction (Wang et al., 2020) indicated that the mutMP mutation compromises a ubiquitination site at the amino acid residue K250 and abolishes four phosphorylation sites at the residues S258, T261, S265, and S267 (Figure S6). Interestingly, phosphorylation is known to regulate the ubiquitin-mediated degradation of viral (Héricourt et al., 2000) and host proteins (Liu et al., 2009). Therefore, the complete lack of the ubiquitination and phosphorylation sites in the “new” C-terminus of mutMP may stabilize mutMP by rendering it less susceptible to the ubiquitin/proteasome system (UPS) of the cell.

In addition, phosphorylation of the MP residues S258, T261, and S265 was demonstrated directly and suggested to represent a mechanism to sequester and functionally inactivate MP (Citovsky et al., 1993); this notion is consistent with the observations that the C-terminal phosphorylation sites are dispensable for the cell-to-cell movement of MP in *N*. *benthamiana* [not shown and (Trutnyeva et al., 2005)]. Thus, deletion of the C-terminal domain may in fact further activate the protein and contribute to the altered function of mutMP.

The requirement for CP for their systemic movement has become an exclusive rule for most plant viruses (Hipper et al., 2013). A unique exception is the members of the genus *Umbravirus*, which naturally lack a CP-encoding gene but are systemically infectious in the form of RNP complexes (Ryabov et al., 2001; Taliansky et al., 2003). Similarly, viroids represent such an exception for subviral agents which often exist in association with helper viruses, e.g., alpha-satellites can replicate their own genomes but depend on their helper begomoviruses for systemic infection (Badar et al., 2021); and tombusvirus-like associated RNAs are capable of autonomous replication but also depend on a virus of the genus *Polerovirus* as a helper for systemic movement and aphid transmission (Passmore et al., 1993). In our experimental system of the CP-deficient TMV genome incapable of systemic transport, we identified a subviral agent capable of replication and CP-independent systemic movement.

### Adaptive virulence conferred by mutMP

Hypersensitive reaction (HR) is a typical response upon recognition by the host resistance (R) proteins of their corresponding viral factor, e.g., HR in response to the N protein against the helicase domain of the TMV replicase protein (Tran et al., 2014). When the R proteins are absent or insufficiently induced, systemic necrosis appears at later stages of infection (Abebe et al., 2021; Roshan et al., 2018). *N*. *benthamiana* does not carry the *N* gene but still develops a TMV-induced necrosis (Guo et al., 2015) which likely inhibits the virus multiplication (Komatsu et al., 2010). We observed that the local necrosis response in *N*. *benthamiana* against TMV is more severe in the absence of CP but is suppressed by the recovery mutant pTMVΔCPmutMP or the presence of CP. Thus, besides gaining the function of CP in systemic movement per se, mutMP also evolved to exhibit a CP-like virulence function in mitigating the host immune response.

Interactions between plants and viruses usually result in the accumulation of SA, JA, or ET (Carr et al., 2010). In *N*-mediated resistance against TMV, ET is highly accumulated and accelerates the HR (Knoester et al., 2001; Ohtsubo et al., 1999). The involvement of an ET-induced transcription factor in the resistance of *N*. *benthamiana* against TMV (Zhu et al., 2022) suggests that the ET signaling pathway also functions in the absence of the *N* gene. Expression profiling of signaling-related genes demonstrated that both mutMP and CP may act as counter-defense factors to suppress the components of the ET signaling pathway, but not of the JA or SA pathways, in local leaves, i.e., to downregulate the ET signaling positive regulators *ACO1*, *EIN2*, and *EIN3*, and to upregulate the negative regulator *ETR1*. These activities of mutMP could contribute to the delayed local necrosis and maintain conditions conducive to virus replication, as indicated by the higher local TMV RNA accumulation and successful systemic infection of pTMVΔCPmutMP.

The host plant can further restrict the cell-to-cell movement of the virus from the infected into the neighboring uninfected cells during the viral approach to the leaf vein, representing yet another defense layer that prevents the viral systemic infection (Nyalugwe et al., 2016). Our analyses indicate differential transcriptional reprogramming of phloem-associated factors by the viruses with different abilities to move systemically. For example, pTMVΔCP—that fails to move systemically—induces the expression of *PLM1*, a callose-independent negative regulator of phloem transport, and of callose synthase genes *CALS3* and *CALS8* whose overexpression can block the phloem transport (Vatén et al., 2011; Yan et al., 2019). In contrast, pTMVΔCPmutMP and pTMV—that spread systemically—suppress the induction of these regulators and strongly downregulates *CALS7*, another callose synthase gene responsible for callose deposition in the phloem (Xie et al., 2011). The effects of mutMP and CP on the host phloem loading/unloading factors do not always parallel each other; for instance, the expression of a cell wall-associated and cadmium-induced glycine-rich protein cdiGRP was dramatically suppressed by pTMVΔCPmutMP but not by pTMV. Taken together, our data suggest that the adaptation of a CP-deficient TMV virus in the *N*. *benthamiana* host creates a multifunctional mutMP that retains its cell-to-cell movement function and gains, at least in part, the function of CP to promote systemic movement and suppress host immunity.

In summary, we propose a model for the TMV-host plant interactions that occur when the systemic movement capacity is lost in pTMVΔCP and regained in pTMVΔCPmutMP. The loss of CP restricts the ability of TMV to move systemically and induces the ET signaling and local necrosis. The lack of CP in pTMVΔCP also increases the expression of phloem-associated resistance factors that regulate the processes of phloem loading and unloading. In contrast, the gain-of-function mutMP mutant is systemically movable and increases the viral RNA accumulation. The mutMP mutant virus accumulates at a higher level and gains a CP-like virulence suppressing the host defenses, e.g., local necrosis, ET pathway, and phloem-associated resistance factors.

### Limitations of the study

This study focuses on the CP-independent systemic transport of defective TMV mutants infecting *N*. *benthamiana*. The spectrum of this ability should be confirmed with other defective tobamoviruses or in additional natural hosts of TMV. Also, only some of the most relevant resistance signaling pathways are characterized in this study. Therefore, a high-throughput transcriptomic or proteomic analysis is necessary for the full understanding of the systemic changes of host signaling pathways suppressed by the pTMVΔCPmutMP gain-of-function mutant.

## STAR★Methods

### Key resources table

**Table:** 

REAGENT or RESOURCE	SOURCE	IDENTIFIER
**Antibodies**		

Rabbit anti-MP antibody	Alpha Diagnostic	#TMVMP11-A
Goat anti-rabbit antibody	Abcam	#ab2057181

**Bacterial and virus strains**		

*Escherichia coli* DH10B	Invitrogen	#EC0113
*Agrobacterium tumefaciens* EHA105	GoldBio	#CC-108-5x50

**Chemicals, peptides, and recombinant proteins**		

TRIzol	Invitrogen	#15596026
NEB2 buffer	NEB	#B7002S
pfu ultra II DNA polymerase	Agilent	#600674
T4 DNA polymerase	Thermo Scientific	#EP0061
BSA	NEB	#B9001
dATP	Thermo Scientific	#R0141
dTTP	Thermo Scientific	#R0171
BP clonase	Invitrogen	#11789100
LR clonase	Invitrogen	#11791020
MgCl_2_	Sigma-Aldrich	#63068–250G
MES	Sigma-Aldrich	#69889–10G
Acetosyringone (3′,5′-Dimethoxy-4′-hydroxyacetophenone)	Sigma-Aldrich	#D134406-1G
Tris base	Sigma-Aldrich	93362–250G
SDS	Sigma-Aldrich	71725–50G
Bromophenol blue	Sigma-Aldrich	114391-5G
DTT	Sigma-Aldrich	43815-1G
PVDF membrane	Immobilon	#IPVH00010
Ponceau S	Sigma-Aldrich	#P7170
Casein	Sigma-Aldrich	#C8654-500G
TBS-T	Sigma-Aldrich	#T9039-10PAK
Kanamycin	British Pharmacopoeia	#BP861
Spectinomycin	Sigma-Aldrich	#S4014-5G
LB broth	Sigma-Aldrich	#L3022-250G

**Critical commercial assays**		

RevertAid Revert Transcription Kit	Thermo Scientific	#K1691
PowerUp SYBR Green Master Mix	Applied Biosystems	#A25741
Opti-4CN Substrate Kit	Bio-Rad	#1708235

**Experimental models: Organisms/strains**		

*Nicotiana benthamiana*	Our laboratory collection	N/A

**Oligonucleotides**		

Primers used for DNA amplification, molecular cloning, and detection, see Table S1	This study	Table S1

**Recombinant DNA**		

pTMVΔCP	Addgene	#80082
pTMVΔCP G	Addgene	#80083
pTMVΔCPmutMP	This study	N/A
pTMVΔCPmutMP2	This study	N/A
pTMV	This study	N/A
pDONR207	Invitrogen	#12213013
pRCS2-PZP CFP-N1-DEST	Our laboratory collection	N/A
pRCS2-PZP YFP	Our laboratory collection	N/A
pRCS2-PZP PDCB1-mRFP	Our laboratory collection	N/A
pRCS2-PZP BAM1-mRFP	Our laboratory collection	N/A

**Software and algorithms**		

Microsoft Office 365	Microsoft	Version 2206
ImageJ	NIH	https://imagej.nih.gov/ij/
BLAST	NCBI	N/A

### Resource availability

#### Lead contact

Further information and requests for resources and reagents should be directed to and will be fulfilled by the lead contact, Phu-Tri Tran (phutri.tran@stonybrook.edu).

#### Materials availability

Plasmids generated in this study are available from the lead contact.

### Experimental model and subject details

#### Plant growth

*Nicotiana benthamiana* plants were grown on soil in an environment-controlled chamber at 23°C under a 16-h light (100 μmol photons m−2 s−1)/8-h dark cycle. One week after sowing, the seedlings were transplanted into 10 × 10 × 15 cm pots filled with vermiculite. Three-week-old *N*. *benthamiana* plants were used for virus inoculation and agrobacterium-mediated transient expression.

#### Bacterial growth

For the Agrobacterium-mediated transient expression, the cells of *Agrobacterium tumefaciens* strain EHA105 harboring the appropriate plasmids were individually added to 1 mL of LB broth containing appropriate antibiotics for vector selection. After the broth cultures had grown for 24 h at 28°C and 200 rpm, 0.1 mL of each was transferred to 4.9 mL of LB broth containing the same antibiotics and 200 μM acetosyringone. The 5-mL cultures were grown under the same conditions for 16 h before the Agrobacterium cells were collected for inoculation into plants.

### Method details

#### Screening for recovery mutants

To identify a gain-of-function mutant that allows pTMVΔCP to regain the capacity for systemic movement, pTMVΔCP G was transiently expressed in two fully expanded leaves of three-week-old *N*. *benthamiana* plants by Agrobacterium-mediated transient expression. Local infection was validated at 3 dpi by visualization of GFP expression under the 377 nm UV light; thereafter, the systemic leaves were imaged every 7 days for the GFP signal and TMV disease symptoms.

The upper leaves of plants that developed severe symptoms at 14 dpi were harvested and subjected to RNA extraction by the TRIzol reagent (Invitrogen #15596026) according to the manufacturer’s instructions. About 4 μg of RNA was used as a reverse transcription template to synthesize cDNA with the RevertAid Revert Transcription Kit (Thermofisher #K1691) and Hexa-random primers. The resulting DNA was diluted 4 times and used as a template for PCR reactions with gene-specific primers listed in Table S1. The PCR products were used as a template for ligation-independent cloning (Schmid-Burgk et al., 2013), confirmed by DNA sequencing, and analyzed using the Basic Local Alignment Search Tool (BLAST) and the original sequence of the GFP expression cassette as a query.

#### Overlapping PCR to generate inserts for plasmid construction

Overlapping PCR was performed as described with minor modifications (Tran et al., 2021). Briefly, specific fragments were generated by the conventional PCR using the pfu ultra II DNA polymerase (Agilent #600674) according to the manufacturer’s instructions. About 10 ng of the smaller fragment and the equivalent amount (1:1 molar ratio) of the larger fragment were used as a template in a 50-μL of PCR reaction with the pfu ultra II DNA polymerase and the experimentally optimized amplification regimen: 95°C for 2 min for initial denaturation, 25 cycles of amplification (95°C for 20 s, 60°C for 5 min, 72°C for 2 min), and final extension at 72°C for 5 min. The resulting PCR products were gel-purified using the DNA Gel Extraction Kit (Bioneer, Daejeon, South Korea) and utilized as inserts for the ligation-independent cloning.

#### Plasmid construction

The pTMVΔCP and pTMVΔCP G plasmids (Addgene #80082 and #80083, respectively) were s a kind gift from Dr. John Lindbo (Ohio State University) (Lindbo, 2007). These plasmids were used as a backbone to produce the TMV clones pTMVΔCPmutMP, and pTMVΔCPmutMP2. These TMV clones were generated by a ligation-independent cloning (Schmid-Burgk et al., 2013) with minor modifications. Briefly, the vector backbone, identical for all clones, and clone-specific individual inserts were amplified by PCR or overlap PCR, respectively, as described (Tran et al., 2021), using the pfu ultra II DNA polymerase (Agilent #600674) and primers listed in Table S1. After gel-purification, 186 ng of the vector DNA or two-fold molar equivalent of each insert DNA was added to a 10-μL chew-back reaction (1X NEB2 buffer, 0.1 unit/μL T4 DNA polymerase, 0.1 μg/μL BSA, and 1 mM dTTP for vector or 1mM dATP for each insert). The reactions were allowed to proceed for 5 min at 27°C, 5 min at 4°C, and 20 min at 75°C. The resulting T-chewed vector (5 μL) of the A-chewed insert (5 μL) were combined and incubated for 30 min at 55°C and overnight at 25°C, followed by transformation into the *E*. *coli* strain DH10B. All generated clones were confirmed by PCR and DNA sequencing.

To construct the CFP-tagged MP variants for assays of cell-to-cell movement and subcellular localization, the MP and mutMP coding sequences were amplified as described above, cloned into pDONR207 by the BP reaction with the Gateway BP Clonase II (Invitrogen #11789100), and transferred into pRCS2-PZP CFP-N1-DEST (Tzfira et al., 2005) by the LR reaction with the Gateway LR Clonase II (Invitrogen #11791020), resulting in constructs expressing MP-CFP or mutMP-CFP, in which the CFP tag is fused to the C-terminus of MP or mutMP.

#### Agrobacterium-mediated transient expression

The plasmid constructs to be tested were transformed into the *A*. *tumefaciens* strain EHA105 by the classical calcium chloride method with minor modifications (Lacroix and Citovsky, 2016). Kanamycin or spectinomycin (50 μg/mL) were used for the selection of TMV clones and pRCS2-PZP-based clones, respectively. Agroinfiltration was conducted as described (Tran and Citovsky, 2021) with minor modifications. Briefly, the overnight liquid cultures of Agrobacterium harboring the tested constructs were harvested by centrifugation at 2,000xg and resuspended in the MMA buffer (10 mM MgCl_2_, 10 mM MES pH 5.7, 200 μM acetosyringone) to OD_600_ of 0.1, 0.2, or 0.001 for virus inoculation, subcellular localization, and movement assays, respectively. For subcellular localization, the suspensions of bacteria with the tested constructs were mixed at a 1:1:1 vol/vol ratio with the suspensions of bacteria harboring reference constructs that express free YFP (a nucleocytoplasmic marker), PDCB1-mRFP (a plasmodesmal marker), or BAM1-mRFP (a plasma membrane marker). These cell mixtures were infiltrated in two abaxial sides of fully expanded leaves of three-week-old *N*. *benthamiana*.

To monitor the expression of pTMVΔCP G, the inoculated plants were periodically imaged using a digital camera with a UV filter under the 377 nm UV light in a dark room. Subcellular localization of MP-CFP was recorded at 2 dpi under a laser scanning confocal microscope (LSM 900, Zeiss) with a 40× objective lens and CFP-, YFP-, and mRFP-specific filters. The cell-to-cell movement of MP-CFP was scored at 2 dpi as multi-cell clusters by counting them under a confocal microscope with a 10× objective lens and a CFP-specific filter.

#### Quantitative RT-PCR (qRT-PCR)

To quantify viral RNA accumulations and transcriptional expression of the host genes, total RNA from 50 mg of the green leaf tissue around the inoculation site, or from an uninoculated, systemic leaf if so indicated, was extracted by the TRIzol reagent and utilized as a reverse transcription template to synthesize cDNA using the RevertAid Revert Transcription Kit and Hexa-random primers. Quantitative PCR (qPCR) was performed as described (Tran and Citovsky, 2021), using a QuantStudio™ 3 real-time PCR system (Applied Biosystems #A28567) and the PowerUp SYBR Green Master Mix (Applied Biosystems #A25741) with the cycling regimen recommended by the manufacturer and gene-specific primers listed in Table S1. Fold change in gene expression is normalized to an internal control gene (Livak and Schmittgen, 2001) for which we utilized the *N*. *benthamiana F-BOX* gene (Liu et al., 2012). Fold change for each condition was calculated by the delta-delta Ct (cycle threshold, i.e., the number of PCR cycles required for the signal to become detectable above the background) method as described (Livak and Schmittgen, 2001; Tran et al., 2018). The resulting fold change was expressed relative to that in the pTMVΔCP-inoculated plants, which was set to 1.0; pTMVΔCP is the parental strain of pTMVΔCPmutMP, which represents the reference point for the pTMVdelCPmutMP movement and serves as a control for possible effects of agroinoculation.

#### Western blotting

To detect the accumulation of the MP or mutMP proteins, total proteins from 100 mg of green leaf tissue around the inoculation site, or from an uninoculated, systemic leaf if so indicated, were extracted by grinding and heating in 0.5 mL of 1X sample buffer (50 mM Tris-Cl pH 6.8, 2% SDS, 0.1% bromophenol blue, 10% glycerol, 100 mM DTT) for 10 min at 95°C, followed by centrifugation at 11,000xg for 1 min. The supernatant (15 μL) was resolved by SDS polyacrylamide gel electrophoresis (PAGE) on a 10% gel and electro-blotted onto PVDF membranes (Immobilon #IPVH00010). The ∼55-kDa large unit of Rubisco was used as the loading control and visualized on the blots by the Ponceau S (Sigma-Aldrich #P7170) staining. The blots were then blocked by casein (1%, pH 8 in the TBS-T buffer), probed with rabbit anti-MP antibody (Alpha Diagnostic #TMVMP11-A, 1:10,000 dilution), followed by the horseradish peroxidase-conjugated goat anti-rabbit antibody (Abcam #ab2057181, 20,000 dilution). The probed blots were analyzed using an Opti-4CN Substrate Kit (Bio-Rad #1708235). The western blot band intensity was quantified by densitometry and MP accumulation was interpreted as pixel value using ImageJ software (https://imagej.nih.gov/ij/) (Schneider et al., 2012).

#### Microsomal extracts and viral RNA immunoprecipitation and quantification

For microsomal extracts, the inoculated or uninoculated, systemic leaves from *N*. *benthamiana* plants with viral symptoms were fixed in 1% formaldehyde (plus 0.01% TrixonX-100) for 10 min in a vacuum. The fixation was stopped by replacing the formaldehyde solution with 125 mM glycine supplemented with 0.01% TrixonX-100 and vacuum treatment for 5 min. The leaves were then washed 4 times with distilled water and dried with absorbent papers. The microsome fraction from the leaves was extracted as described (Abas and Luschnig, 2010) and resuspended by sonication in the microsome protein solubilization buffer (100mM Tris-HCl pH 7.3, 150 mM NaCl, 1mM EDTA, 10% glycerol, 20 mM NaF, 1% Triton X-100, 1mM PMSF, complete protease inhibitor cocktail 1X). Finally, the microsome extracts were diluted 10 times in the dilution buffer (16.7 mM Tris/HCl pH 8.0, 167 mM NaCl, 1.1% w/v Triton X-100, 1.2 mM EDTA pH 8.0, 0.1 mM PMSF) whereas an undiluted aliquot of the original microsomal extract (10 μL) was stored at −80°C for use as input control.

Viral RNA immunoprecipitation was performed as described (Terzi and Simpson, 2009) with minor modifications. Briefly, for each immunoprecipitation, 5 μL of anti-MP antibody (Alpha Diagnostic #TMVMP11-A) was immobilized on 50 μL of Protein A Sepharose beads (Invitrogen #101041) for overnight at 4°C in the IP buffer (15 mM Tris/HCl pH 8.0, 150 mM NaCl, 1% w/v Triton X-100, 1 mM EDTA pH 8.0, 0.1 mM PMSF). The anti-MP beads were added to 600 μL of the diluted microsomal extract and incubated overnight at 4°C with gentle orbital shaking. Then, the supernatants were removed, and the beads were washed twice for 5 min on ice with washing buffer 1 (20mM Tris/HCl pH 8.0, 150 mM NaCl, 0.1% w/v SDS, 1% w/v Triton X-100, 2 mM EDTA pH 8.0), washing buffer 2 (20 mM Tris/HCl pH 8.0, 500 mM NaCl, 0.1% w/v SDS, 1% w/v Triton X-100, 2 mM EDTA pH 8.0), washing buffer 3 (10 mM Tris/HCl pH 8.0, 250 mM LiCl, 1% w/v Igepal CA-630, 1 mM EDTA pH 8.0, 1% w/v sodium deoxycholate), and washing buffer 4 (10 mM Tris/HCl pH 8.0, 1 mM EDTA pH 8.0). The beads or the input control were combined with 0.5 mL of the TRIzol reagent (Invitrogen #15596026), incubated at 55°C for 5 min to reverse crosslinking, followed by RNA extraction (Marmisollé et al., 2018). The cDNAs were synthesized from 50 ng of the extracted RNAs using the RervertAid RT kit (Thermofisher, #K1691) and analyzed by qPCR using *MP*-specific primers (see Table S1) and a QuantStudio™ 3 Real-Time PCR System (Applied Biosystems #A28567) with the PowerUp SYBR Green Master Mix (Applied Biosystems #A25741). Fold enrichment for each reaction was normalized to the input total viral RNA and calculated by the delta-delta Ct method as described (Marmisollé et al., 2018) relative to the total accumulated MP determined by western blot densitometry.

### Quantification and statistical analysis

All representative images reflect a minimum of three biological replicates. All quantitative data were derived from the numbers of biological replicates indicated for each specific experiment in the Figure legends. Statistical significance of differences in sample means was evaluated by the two-tailed t-test using Excel 365 (Microsoft) software, with P-values < 0.05, 0.01, 0.001, or 0.0001 corresponding to the statistical probability of >95%, 99%, 99.9%, or 99.99% respectively, considered statistically significant.

## Data Availability

Individual data points of quantitative graphs are available in Tables S2–S10. This paper does not report original code. Any additional information required to reanalyze the data reported in this paper is available from the lead contact upon request (phutri.tran@stonybrook.edu).
