# Supplementary material for: Gain-of-function mutant of movement protein allows systemic transport of a defective tobacco mosaic virus
Source: iScience. 2022 Nov 3;25(12):105486. doi: 10.1016/j.isci.2022.105486 (PMC9676214; doi:10.1016/j.isci.2022.105486)
Supplement: Document S1. Figures S1–S7 and Tables S1–S11 [file mmc1.pdf]

iScience, Volume *25*

Supplemental information

Gain-of-function mutant of movement protein allows systemic transport of a defective tobacco mosaic virus

Phu-Tri Tran, Mi-Sa Vo Phan, and Vitaly Citovsky

SUPPLEMENTARY INFORMATION

Supplementary Figures with Legends

Fig. S1. Local nucleotide map of TMV∆CPmutMP mutants, structure of the TMV genome, and relative accumulation of the viral genomic RNA and 3'UTR RNA in *N. benthamiana* plants inoculated with the wild-type TMV and TMV∆CP and TMV∆CPmutMP mutants, related to Fig. 1B. (A) Nucleotide sequences flanking the mutMP and mutMP2 mutations in TMV∆CP, TMV∆CPmutMP, and TMV∆CPmutMP2. The ruler indicates the nucleotide positions n 5,600 to 5,800 relative to the TMV genome; dashes "-" indicate the deleted nucleotides in the mutants. (B) A schematic diagram of the TMV strain U1 genome structure and its cis-acting elements surrounding the mutMP mutation. The locations of the indicates major coding sequences and cis-acting genome elements are based on the following detailed information (Grdzelishvili et al., 2000; van Belkum et al., 1985; Zeenko et al., 2002):

TMV ssRNA (1-6395 nucleotides, nt) (accession number NC_001367.1).

RdRp (RNA-directed RNA polymerase) sequence includes the 183K protein gene (69-4919 nt; at 3417-3419 nt there is a readthrough codon) and the 126K protein gene (69-3419 nt).

MP gene (4903-5709 nt); transcription start site at 4838 and translation start site at 4903,

Full MP sg prom (4743-4878 nt; on the negative strand).

Minimal MP subgenomic (sg) promoter (prom) (4803-4848 nt; on the negative strand).

MP sg prom stem-loop SL1 structure (4807-4827 nt, positive regulator).

MP sg prom stem-loop SL2 structure (4755-4801 nt, negative regulator).

CP gene (5712-6191 nt); transcription start site at 5642 nt and translation start site at 5713 nt.

Full CP sg prom (5485-5696 nt; on the negative strand).

Minimal CP sg prom (5573-5654 nt; on the negative strand).

CP sg prom stem-loop SL1 structure (5543-5694 nt).

3'UTR (6178-6395 nt); the 3'UTR sequence overlaps the last 14 nt of the CP gene.

3'UTR upstream pseudoknot (PK) domain (UPD) (6217-6290 nt); includes 3 PKs (PK1, PK2, PK3).

3'UTR tRNA-like structure (TLS) (6291-6395 nt).

The mutMP mutation lost the genomic MP sequence 5659-5706 nt.

RdRp-specific amplicon, MP-specific amplicon, and 3'UTR-specific amplicons indicate the location of the qRT-PCR-amplified sequences that detect the viral genomic RNA, the viral total RNA (i.e., the genomic RNA and the RNA produced from the MP subgenomic promoter, respectively), and the 3'UTR-specific RNA produced mainly from the CP subgenomic promoter. (C) Relative accumulation of the viral genomic RNA in the inoculated and systemic leaves. The qRT-PCR analysis was performed using the RdRp-specific primers (see Table S1). (D) Relative accumulation of the viral 3'UTR RNA in the inoculated and systemic leaves. The qRT-PCR analysis was performed using the 3'UTR-specific primers (see Table S1). Left panels. Inoculated leaves at 4 dpi. Right panels. Systemic leaves at 14 dpi. Viral RNA accumulation in leaves inoculated with pTMV∆CP was set as 1.0. Individual data points are represented by black dots and their numerical values are listed in Table S8. Differences between mean values assessed by the two-tailed t-test are statistically significant for the p-values * $p < 0.05$, ** $p < 0.01$, and *** $p < 0.001$; $p \geq 0.05$ are not statistically significant (ns).

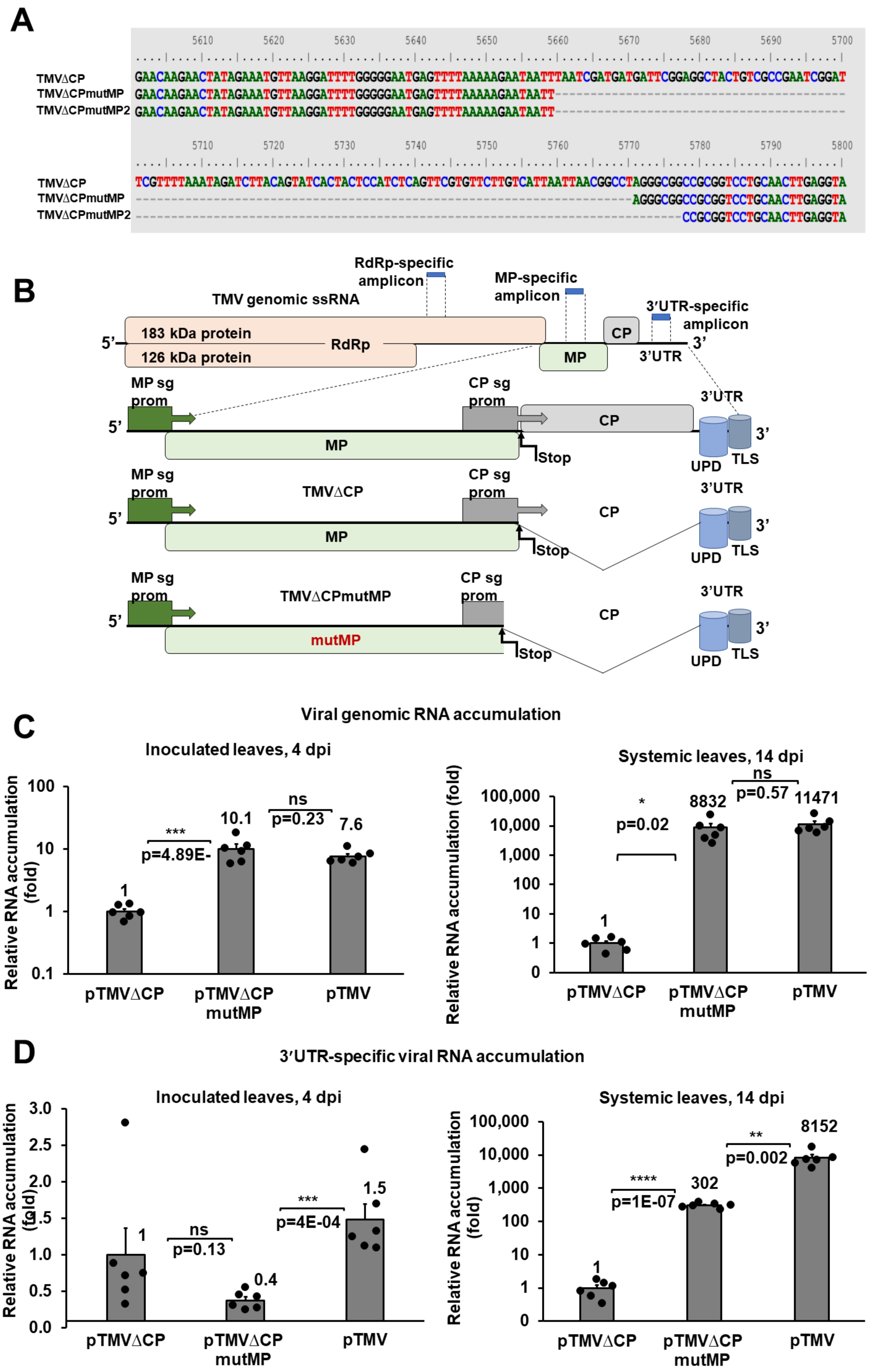
A
TMVΔCP
TMVΔCPmutMP
TMVΔCPmutMP2
B
RdRp-specific amplicon
MP-specific amplicon
3′UTR-specific amplicon
TMV genomic ssRNA
183 kDa protein
126 kDa protein
RdRp
CP
MP
3'UTR
5'
3'
MP sg prom
CP sg prom
Stop
UPD
TLS
TMVΔCP
TMVΔCPmutMP
mutMP
C
Viral genomic RNA accumulation
Inoculated leaves, 4 dpi
Systemic leaves, 14 dpi
Relative RNA accumulation (fold)
***
p=4.89E-
ns
p=0.23
*
p=0.02
ns
p=0.57
1
10.1
7.6
8832
11471
pTMVΔCP
pTMVΔCP mutMP
pTMV
D
3′UTR-specific viral RNA accumulation
Inoculated leaves, 4 dpi
Systemic leaves, 14 dpi
Relative RNA accumulation (fold)
ns
p=0.13
***
p=4E-04
****
p=1E-07
**
p=0.002
1
0.4
1.5
302
8152
pTMVΔCP
pTMVΔCP mutMP
pTMV

Fig. S2. Subcellular localization of mutMP coexpressed with different subcellular localization markers, related to Fig. 2B. The images were recorded using confocal microscopy with a 40X objective lens and CFP, YPF, and RFP filters. Autofluorescence and free YFP were used to indicate cell viability and chloroplastic and nucleocytoplasmic localization, respectively. (A) Co-expression of TMV MP-CFP or TMV mutMP-CFP with the plasmodesmal marker PDCB1-mRFP. (B) Co-expression of TMV MP-CFP or TMV mutMP-CFP with the plasma membrane marker BAM1-mRFP. (C) Co-expression of TMV MP-CFP or TMV mutMP-CFP with the ER membrane marker ER-mRFP. (D) Co-expression of TMV MP-CFP or TMV mutMP-CFP with the cell wall marker PRP1-mRFP. Images are single confocal sections.

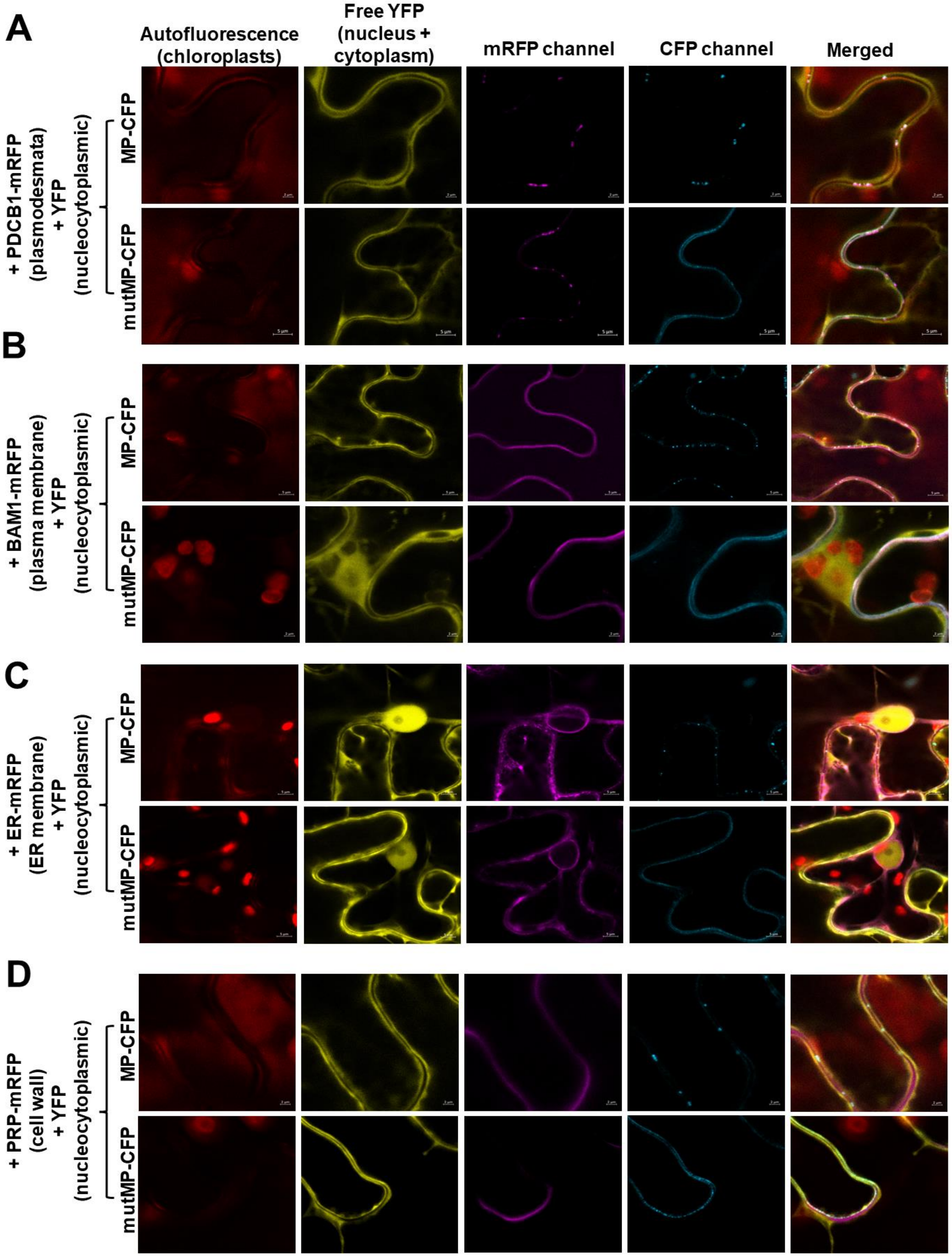
A
Autofluorescence (chloroplasts)
Free YFP (nucleus + cytoplasm)
mRFP channel
CFP channel
Merged
+ PDCB1-mRFP (plasmodesmata) + YFP (nucleocytoplasmic)
MP-CFP
mutMP-CFP
B
+ BAM1-mRFP (plasma membrane) + YFP (nucleocytoplasmic)
MP-CFP
mutMP-CFP
C
+ ER-mRFP (ER membrane) + YFP (nucleocytoplasmic)
MP-CFP
mutMP-CFP
D
+ PRP-mRFP (cell wall) + YFP (nucleocytoplasmic)
MP-CFP
mutMP-CFP

Fig. S3. Systemic viral disease symptoms developed by pTMV∆CP, pTMV∆CPmutMP, and pTMV in *N. benthamiana*, related to Fig. 3A. Upper leaves (with older-to-younger numerical order indicated) were detached and photographed at 14 dpi.

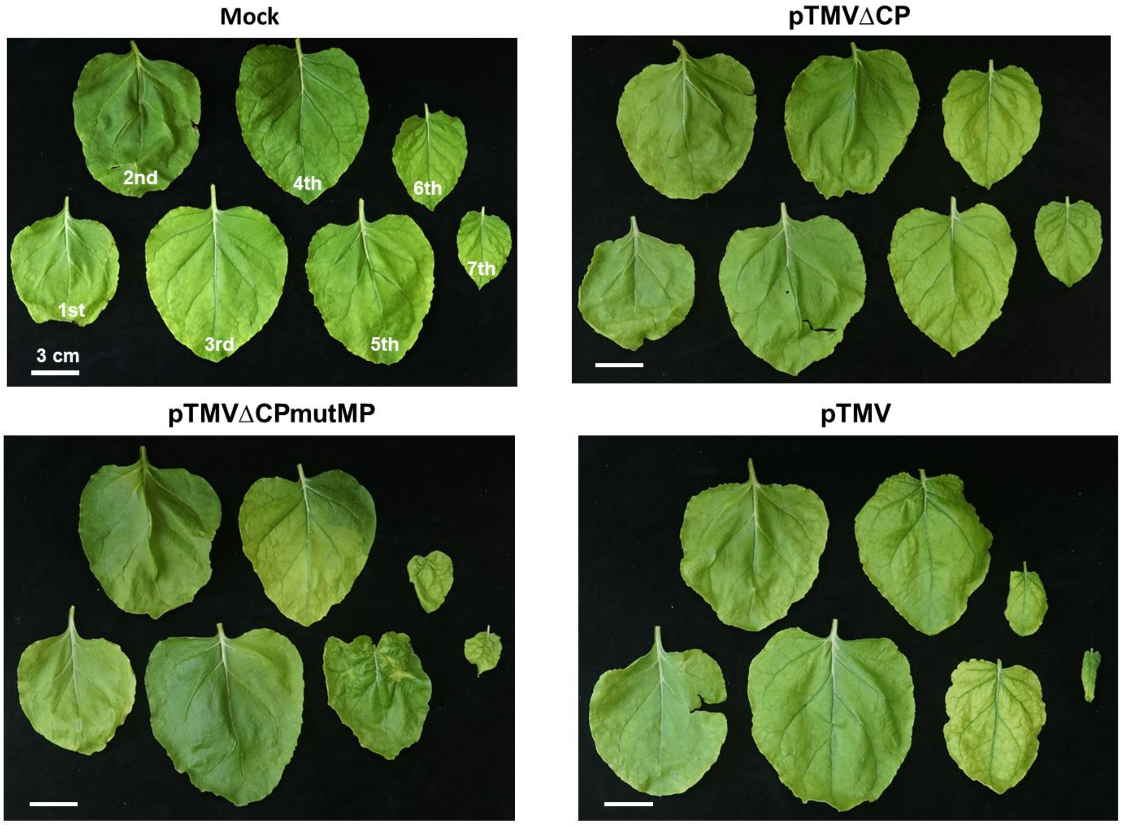

Fig. S4 Accumulation of MP-associated viral RNA in the microsome fraction of systemic *N. benthamiana* leaves relative to the total accumulation of MP, related to Fig. 3C. (A) A schematic diagram of the main technical steps of immunoprecipitation of the MP-associated viral RNA and total MP using the anti-MP-antibody and analysis of the resulting data. (B) Relative viral RNA enrichment in the microsome fraction of systemic leaves immunoprecipitated with the anti-MP antibody and analyzed by qRT-PCR. MP-associated viral RNA accumulation was calculated relative to the total accumulated MP. Accumulation of MP-associated viral RNA in leaves inoculated with pTMV was set as 1.0. Numerical values for individual data points are listed in Table S9.

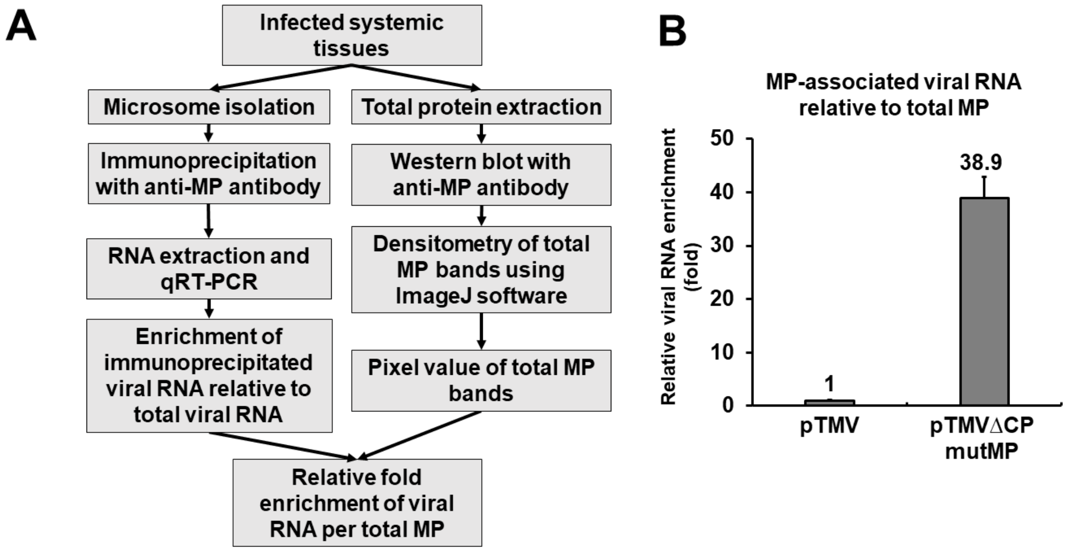

Fig. S5. TMVΔCPmutMP suppresses the expression of the mitochondrial *ISE1* and chloroplastic *ISE2*, genes related to Fig. 5. Expression levels of *ISE1* and *ISE2* were determined by the qRT-PCR analysis of the inoculated *N. benthamiana* leaves at 4 dpi with the MMA buffer (Mock), pTMVΔCP, pTMVΔCPmutMP, or pTMV. Individual data points are represented by black dots and their numerical values are listed in Table S10. Differences between mean values assessed by the two-tailed t-test are statistically significant for the p-values * $p < 0.05$ and ** $p < 0.01$.

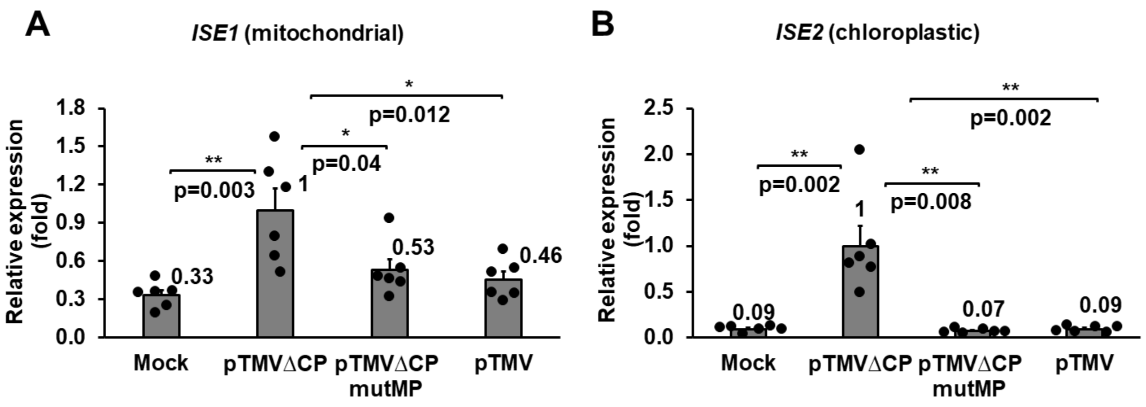

Fig. S6. Predicted post-translational modification motifs at the C-terminus of MP and mutMP, related to figure 3C. The prediction analysis was performed using the MusiteDeep learning network (https://www.musite.net).

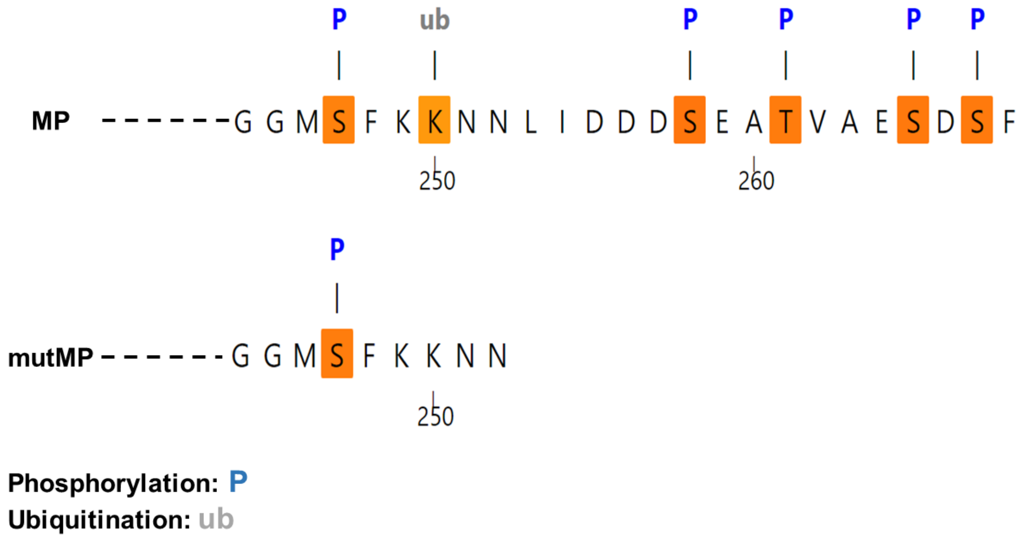

Fig. S7. Accumulation of viral MP in the inoculated and systemic leaves of *N. benthamiana*: raw data, related to Fig. 3C. (A) Inoculated leaves. (B) Systemic leaves. The inoculated leaves at 4 dpi and systemic leaves at 14 dpi were collected from plants inoculated with the MMA buffer (Mock), pTMVΔCP, pTMVΔCPmutMP, or pTMV. Total cell proteins were extracted, resolved by SDS-PAGE, and electroblotted onto a PVDF membrane. The blots were stained with Ponceau S (left panels) and subjected to western blot analysis using the anti-MP antibody (right panels).

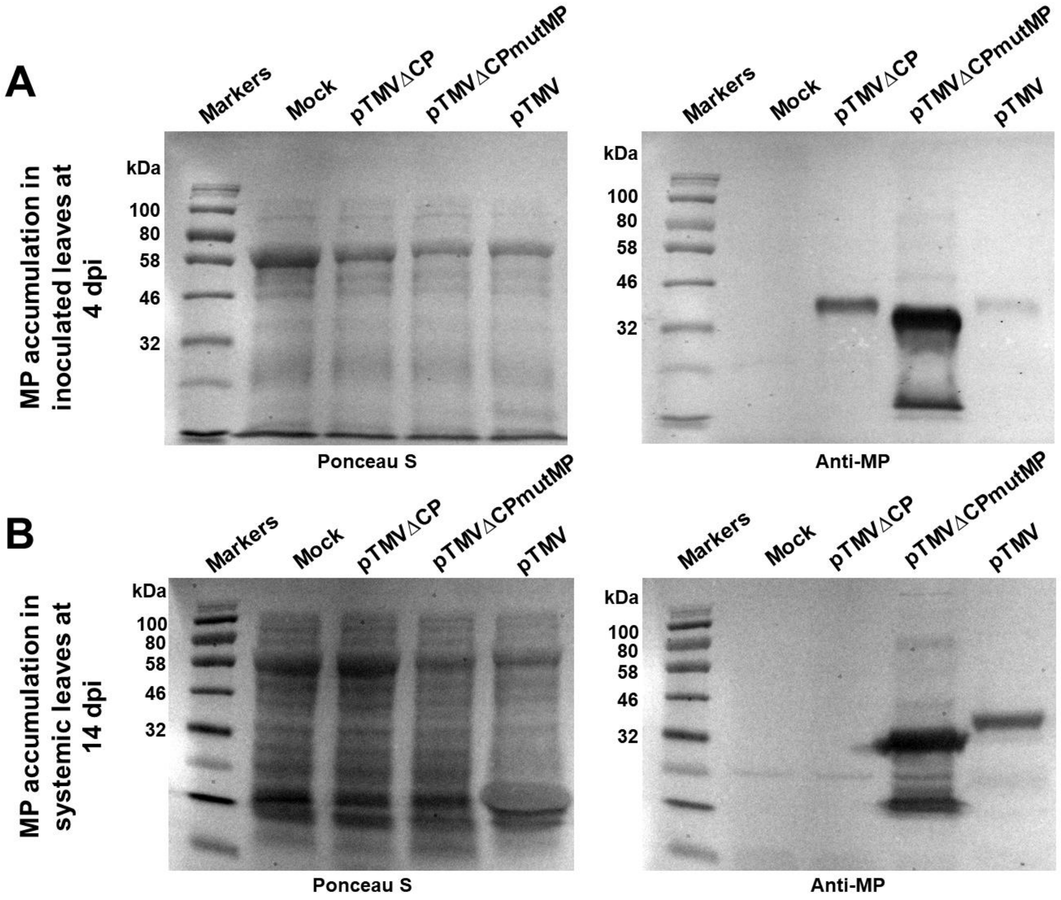

Supplementary Tables

Table S1. Primers used for DNA amplification, molecular cloning, and detection, related to STAR Methods.

No.	Primer name	Sequence (5' to 3')	Accession	Purpose
1	pTMV vector Fw[a]	ggtcctcgccggcggtttttcg	Addgene plasmid #80082	Amplify the 7,405-bp vector fragment
2	pTMV vector Rv[a]	tctgcgctggtgtctttcttgtg	Addgene plasmid #80082	Amplify the 7,405-bp vector fragment
3	pTMV insert Fw	cacaagaaagacaccagcgcaga	Addgene plasmid #80082	Overlapping PCR to amplify insert fragments
4	pTMV Insert Rv	gacgaccaagaagcgaaaaaccgc	Addgene plasmid #80082	Overlapping PCR to amplify insert fragments
5	pTMVΔCP 6610 Rv	gcgatccaagacacaaccct	Addgene plasmid #80082	PCR1 with primer No. 3 for mutants pTMVΔCPmutMP or pTMVΔCPmutMP2
6	pTMV 3UTR Fw	ggtcctgcaacttgaggtagtca	Addgene plasmid #80082	PCR2 with primer No. 4 for mutants pTMVΔCPmutMP or pTMVΔCPmutMP2
7	attB1 TMV MP Fw	ggggacaagtttgtacaaaaaagcaggctcaatggctctagttgttaaaggaaaagtg	Addgene plasmid #80082	PCR and clone MP in pDONR207
8	attB2 TMV MP Rv	ggggaccactttgtacaagaaagctgggtgaaacgaatccgattcggcga	Addgene plasmid #80082	PCR and clone MP in pDONR207
9	AttB2 mutMP Rv	ggggaccactttgtacaagaaagctgggtgattattctttttaaaactcattccCccaa	Addgene plasmid #80082	PCR with primer No. 11 and clone mutMP in pDONR207
10	TMV MP 552 Fw	agggcccatggaacttacag	Addgene plasmid #80082	qPCR for detection of viral RNA, specific for MP
11	TMV MP 670 Rv	tccctttgcggacatcactc	Addgene plasmid #80082	qPCR for detection of viral RNA, specific for MP
12	TMV 3513 Fw	aagtgtctcccaggcaacag	Addgene plasmid #80082	qPCR for detection of viral RNA, specific for RdRp
13	TMV 3637 Rv	ttaggcgcagcaacagactt	Addgene plasmid #80082	qPCR for detection of viral RNA, specific for RdRp
14	TMV 5840 Fw	acacgtggtgcgtacgataa	Addgene plasmid #80082	qPCR for detection of viral RNA, specific for 3′UTR

15	TMV 5960 Rv	gctttattacgtgcctgcgg	Addgene plasmid #80082	qPCR for detection of viral RNA, specific for 3′UTR
16	F-Box Fw	tttgctcctccaagatggca	Nbv6.1trP36478 (http://benthgenome.qut.edu.au/)	Internal control for qPCR
17	F-Box Rv	acctgggaggcatcctgcttat	Nbv6.1trP36478	Internal control for qPCR
18	NPR1 Fw	gactacttcgccgacgctaa	Nbv6.1trP57501	qPCR for detection of *NPR1*
19	NPR1 Rv	ctggcctacaagccacatga	Nbv6.1trP57501	qPCR for detection of *NPR1*
20	EIN2 Fw	ttcaaattcccattggccgc	Nbv6.1trP49428	qPCR for detection of *EIN2*
21	EIN2 Rv	cctggttgcctactggctta	Nbv6.1trP49428	qPCR for detection of *EIN2*
22	COI1 Fw	aaggtgccccaacttggaaa	Nbv6.1trP36054	qPCR for detection of *COI1*
23	COI1 Rv	cgagcaaaaccagccgaaaa	Nbv6.1trP36054	qPCR for detection of *COI1*
24	ACO1 Fw	aatgtccgcgccctgaatta	Nbv6.1trP32986	qPCR for detection of *ACO1*
25	ACO1 Rv	aaatgatggcctcaccagca	Nbv6.1trP32986	qPCR for detection of *ACO1*
26	ETR1 Fw	aagtttgcggaaaaccaggc	Nbv6.1trP54888	qPCR for detection of *ETR1*
27	ETR1 Rv	tgcaggcaatctgggcataa	Nbv6.1trP54888	qPCR for detection of *ETR1*
28	EIN3 Fw	ggcgtggaaagttggtgttc	Nbv6.1trP71204	qPCR for detection of *EIN3*
29	EIN3 Rv	acaccgtcaacgtcgtactc	Nbv6.1trP71204	qPCR for detection of *EIN3*
30	NbPLM1 Fw	gagctccgttcagctatccc	Nbv6.1trP35848	qPCR for detection of *PLM1*
31	NbPLM1 Rv	aagaccaggagcttcaaccg	Nbv6.1trP35848	qPCR for detection of *PLM1*
32	NbGSD1 Fw	ccgcagccctacttcttcaa	Nbv6.1trP68046	qPCR for detection of *GSD1*
33	NbGSD1 Rv	caagcagctgccctttttagc	Nbv6.1trP68046	qPCR for detection of *GSD1*
34	NbcdiGRP Fw	ccgagggtcgattggaaaca	Nbv6.1trP20803	qPCR for detection of *cdiGRP*
35	NbcdiGRP Rv	tcaccgccaacgtcattgta	Nbv6.1trP20803	qPCR for detection of cdiGRP
36	NbCals3 Fw	tttgaaacttgggcaagccg	Nbv6.1trP32592	qPCR for detection of *CALS3*

37	NbCals3 Rv	cagcaagcatattgggcgac	Nbv6.1trP32592	qPCR for detection of *CALS3*
38	NbCALS7 Fw	gcaagctcgcattcagctac	Nbv6.1trP56034	qPCR for detection of *CALS7*
39	NbCals7 Rv	ccgtaacatccctagcgtcc	Nbv6.1trP56034	qPCR for detection of *CALS7*
40	NbCals8 Fw	tgtgcctgtggttggaaagt	Nbv6.1trP69207	qPCR for detection of *CALS8*
41	NbCals8 Rv	tgctccagcattgctcttga	Nbv6.1trP69207	qPCR for detection of *CALS8*
42	NbISE1 Fw	gacacctggacggatagctg	Nbv6.1trP42855	qPCR for detection of *ISE1*
43	NbISE1 Rv	caaccccaacttcttgctgc	Nbv6.1trP42855	qPCR for detection of *ISE1*
44	NbISE2 Rv	gctgcagtctgtggaagtct	Nbv6.1trP13597	qPCR for detection of *SE2*
45	NbISE2 Rv	aggcgagccaaatctccttc	Nbv6.1trP13597	qPCR for detection of *ISE2*

(a) Fw, forward primers; Rv, reverse primers.

Table S2. Raw data for quantitative analyses of cell-to-cell movement, related to Fig. 2.

	MP-CFP cell clusters			mutMP-CFP cell clusters		
Replicate	1-cell	2-cell	3-cell	1-cell	2-cell	3-cell
#1	40	21	3	45	15	1
#2	56	17	4	21	13	2
#3	32	13	2	29	10	4
#4	53	22	8	69	19	9
#5	50	20	7	27	9	4

Table S3. Raw data for quantification of relative accumulation of the total viral RNA and the MP protein in the inoculated and systemic leaves, related to Fig. 3.

Relative viral RNA accumulation (fold change) in inoculated leaves at 4 dpi			
Replicate	pTMVΔCP	pTMVΔCPmutMP	pTMV
#1	0.90891	8.39577	5.87485
#2	0.98251	16.26094	7.65707
#3	1.07649	6.25355	6.72187
#4	0.88723	9.10252	10.12283
#5	1.07656	19.05246	8.70637
#6	1.06828	12.55554	5.11176
Relative viral RNA accumulation (fold change) in inoculated leaves at 14 dpi			
Replicate	pTMVΔCP	pTMVΔCPmutMP	pTMV
#1	1.95468	30,786.7	58,679.4
#2	1.24267	23,015.7	51,544.3
#3	0.86680	19,131.5	177,645.7
#4	0.88613	26,910.5	25,940.2
#5	0.55977	14,763.3	81,690.2
#6	0.48992	10,896.53	44,266.75

Table S4. Raw data for quantification of the effects of TMVΔCPmutMP on the expression of the salicylic acid (SA) and jasmonic acid (JA) pathways genes, related to Fig. 4B, C.

Relative expression (fold change) of *NPR1*				
Replicate	Mock	pTMVΔCP	pTMVΔCPmutMP	pTMV
#1	0.83999	1.24166	0.71674	1.88888
#2	1.63064	0.94919	0.79386	0.91088
#3	1.07385	1.25978	1.62410	0.54287
#4	1.17665	0.77510	0.22776	0.60039
#5	1.73707	0.55616	0.34026	0.28930
#6	0.85049	1.21808	0.88630	0.00230
Relative expression (fold change) of *COI1*				
Replicate	Mock	pTMVΔCP	pTMVΔCPmutMP	pTMV
#1	1.81818	1.10991	1.2418	1.85349
#2	1.42739	0.7510	0.74385	0.91730
#3	1.76924	0.82590	0.90361	1.45328
#4	1.70658	1.01390	1.02496	1.28790
#5	1.35872	1.15651	1.29946	1.38087
#6	1.03146	1.14274	1.41920	1.76424

Table S5. Raw data for quantification of the effects of TMVΔCPmutMP on the expression of the ethylene (ET) signaling genes, related to Fig. 4D, F, G, H.

Relative expression (fold change) of EIN2				
Replicate	Mock	pTMVΔCP	pTMVΔCPmutMP	pTMV
#1	0.80182	1.42637	0.52604	1.47E-05
#2	2.08255	1.00037	0.27049	0.23795
#3	0.68053	1.11206	0.33920	0.17459
#4	5.97E-06	0.81271	0.33131	0.05192
#5	1.47206	0.60887	0.15470	0.16524
#6	0.46980	1.03959	0.48287	2.57E-05
Relative expression (fold change) of *ACO1*				
Replicate	Mock	pTMVΔCP	pTMVΔCPmutMP	pTMV
#1	0.23226	1.01453	0.63564	0.30621
#2	0.66858	1.31332	0.52086	0.19143
#3	0.77589	1.22344	0.99720	0.38265
#4	0.46266	0.42953	0.67668	0.33979
#5	0.23194	0.78118	0.34268	0.31476
#6	0.34409	1.23797	0.46180	0.27901
Relative expression (fold change) of *EIN3*				
Replicate	Mock	pTMVΔCP	pTMVΔCPmutMP	pTMV
#1	0.45179	1.22618	0.56698	0.51775
#2	0.95987	1.36150	0.31454	0.17403
#3	1.28075	1.26602	0.65570	0.59331
#4	0.77566	0.57282	0.44005	0.36954
#5	0.26830	0.75062	0.21963	0.23208
#6	0.53262	0.82283	0.15816	0.25805
Relative expression (fold change) of *ETR1*				
Replicate	Mock	pTMVΔCP	pTMVΔCPmutMP	pTMV
#1	0.77231	0.82427	5.24811	2.28064
#2	1.07288	1.39617	8.19555	1.98076
#3	1.21439	0.93752	5.98185	2.55543
#4	1.12881	1.20378	4.75704	2.08607
#5	1.05285	0.83280	5.30335	1.55850
#6	0.70874	0.80543	9.35918	1.88972

Table S6. Raw data for quantification of suppression of the host genes involved in phloem loading/unloading by TMVΔCPmutMP, related to Fig. 5B, C, D.

Relative expression (fold change) of *PML1*				
Replicate	Mock	pTMVΔCP	pTMVΔCPmutMP	pTMV
#1	0.30393	1.14324	0.27484	0.27761
#2	0.45023	0.99191	0.34767	0.31825
#3	0.42213	0.83681	0.65199	0.48825
#4	0.39085	0.63330	0.46142	0.54088
#5	0.35588	1.15990	0.38315	0.79696
#6	0.45268	1.23482	0.48109	0.75238
Relative expression (fold change) of *GSD1*				
Replicate	Mock	pTMVΔCP	pTMVΔCPmutMP	pTMV
#1	1.01412	0.79019	0.46650	1.26033
#2	1.28299	1.07318	0.58273	1.70383
#3	1.04118	0.82028	0.55151	0.65633
#4	0.73787	1.21740	0.83215	0.61808
#5	1.39766	0.98115	0.39979	0.36724
#6	1.43303	1.11777	0.51021	0.76878
Relative expression (fold change) of *cdiGRP*				
Replicate	Mock	pTMVΔCP	pTMVΔCPmutMP	pTMV
#1	1.22920	1.28080	0.10178	3.27720
#2	1.71741	1.29361	0.03808	3.69444
#3	0.95411	0.76894	0.14499	1.40856
#4	0.69814	0.90254	0.13026	0.49710
#5	3.46197	1.58018	0.09191	0.26709
#6	1.69960	0.17389	0.05988	0.68025

Table S7. Raw data for quantification of suppression of the callose synthase genes by TMVΔCPmutMP, related to Fig. 5F, G, H.

Relative expression (fold change) of *CALS3*				
Replicate	Mock	pTMVΔCP	pTMVΔCPmutMP	pTMV
#1	0.45489	0.93992	0.43528	0.38299
#2	0.73954	0.72612	0.14906	0.33891
#3	0.66049	0.59803	0.25106	0.23631
#4	0.32649	0.52468	0.39528	0.10287
#5	0.46194	1.15673	0.17422	0.33322
#6	0.46214	2.05449	0.29969	0.30394
Relative expression (fold change) of *CALS8*				
Replicate	Mock	pTMVΔCP	pTMVΔCPmutMP	pTMV
#1	0.23741	0.82554	0.51543	0.31011
#2	0.40987	0.66237	0.24437	0.85802
#3	0.50383	0.89282	0.17748	0.28033
#4	0.34413	0.64961	0.23471	0.23780
#5	0.53244	0.80853	0.21150	0.61210
#6	0.29193	2.16111	0.46826	0.68634
Relative expression (fold change) of *CALS7*				
Replicate	Mock	pTMVΔCP	pTMVΔCPmutMP	pTMV
#1	0.91137	0.35186	0.14352	0.50967
#2	0.99997	0.69009	0.04421	0.60234
#3	1.56999	0.74887	0.06740	0.06408
#4	1.64035	0.85430	0.26107	0.03525
#5	1.29625	1.09181	0.06681	0.02349
#6	0.95049	2.26303	0.15129	0.58806

Table S8. Raw data for quantification of relative accumulation of the viral genomic RNA and the 3'UTR RNA in the inoculated and systemic leaves, related to Fig. S1B, S1C, and Fig. 2B.

Relative viral genomic RNA accumulation (fold change) in inoculated leaves at 4 dpi			
Replicate	pTMVΔCP	pTMVΔCPmutMP	pTMV
#1	0.67884	6.25081	6.31107
#2	0.84027	10.35674	8.36026
#3	0.96011	5.80058	6.71232
#4	1.26650	11.23201	7.51548
#5	1.30837	17.93796	11.00005
#6	0.94591	9.18044	5.92567
Relative viral genomic RNA accumulation (fold change) in systemic leaves at 14 dpi			
Replicate	pTMVΔCP	pTMVΔCPmutMP	pTMV
#1	1.56694	9761.39970	6670.58328
#2	1.41263	8482.89069	7921.59502
#3	0.93364	4734.82277	25975.63386
#4	1.09313	23727.28274	5815.96346
#5	0.41626	3724.55846	13745.18435
#6	0.57741	2563.96177	8698.88143
Relative viral 3'UTR RNA accumulation (fold change) in inoculated leaves at 4 dpi			
Replicate	pTMVΔCP	pTMVΔCPmutMP	pTMV
#1	0.74759	0.43014	1.12247
#2	0.88094	0.55292	2.44117
#3	0.52226	0.25176	1.09335
#4	2.80863	0.44847	1.25013
#5	0.71665	0.30949	1.69222
#6	0.32394	0.27879	1.32802
Relative viral 3'UTR RNA accumulation (fold change) in systemic leaves at 14 dpi			
Replicate	pTMVΔCP	pTMVΔCPmutMP	pTMV
#1	1.79656	385.99312	5544.26141
#2	1.35698	328.31618	7120.93032
#3	0.79602	308.88656	17040.49683
#4	1.13569	225.44828	3965.63065
#5	0.34251	297.15479	8229.68307
#6	0.57223	265.16451	7010.99961

Table S9. Raw data for quantification of accumulation of viral MP-associated RNA in the systemic leaves, related to Fig. S4 and Fig. 3C.

Ct value							
	Input RNA (1:6 v/v dilution)				Precipitated RNA		
Replicate	Water	Mock	pTMV	pTMVΔCPmutMP	Mock	pTMV	pTMVΔCPmutMP
#1	39.8	34	8.77	19.9	33.4	15.6	18.7
#2	38.1	35.2	8.93	20.1	33.7	16	19.1
#3	39	33	8.39	19.9	35.6	15.5	19

Relative accumulate (fold change) of IP per input		
Replicate	pTMV	pTMVΔCPmutMP
#1	0.0014284	0.36672
#2	0.0012330	0.34874
#3	0.0011893	0.29589
**Pixels of detected MP (after background subtraction)**		
Replicate	pTMV	pTMVΔCPmutMP
#1	36.2	239
#2	38.4	233
#3	30.6	233
**RNA enrichment (fold change) per pixel of detected MP**		
Replicate	pTMV	pTMVΔCPmutMP
#1	3.9448E-05	0.0015364
#2	3.2130E-05	0.0014945
#3	3.8842E-05	0.0012675
**Relative RNA enrichment per pixel of detected MP**		
Replicate	pTMV	pTMVΔCPmutMP
#1	1.07	41.74
#2	0.87	40.6
#3	1.06	34.44

Table S10. Raw data for quantification of suppression of the *ISE1* and *ISE2* genes by TMVΔCPmutMP, related to Fig. S5 and Fig. 5.

Relative expression (fold change) of *ISE1*				
Replicate	Mock	pTMVΔCP	pTMVΔCPmutMP	pTMV
#1	0.36315	1.30135	0.93187	0.54312
#2	0.35111	1.17515	0.46435	0.51487
#3	0.18971	0.64266	0.54557	0.35390
#4	0.24766	0.51451	0.43655	0.29116
#5	0.35912	0.79540	0.47948	0.34367
#6	0.48198	1.57093	0.32183	0.69420
Relative expression (fold change) of *ISE2*				
Replicate	Mock	pTMVΔCP	pTMVΔCPmutMP	pTMV
#1	0.03713	0.81161	0.10536	0.11350
#2	0.08771	1.01213	0.06691	0.13493
#3	0.12862	0.76392	0.08752	0.07371
#4	0.08643	0.48995	0.05983	0.06003
#5	0.10781	0.88205	0.05137	0.05090
#6	0.11792	2.04034	0.04252	0.12073
